# Tunable Pnictogen Bonding at the Service of Hydroxide Transport across Phospholipid Bilayers

**DOI:** 10.1021/jacs.4c00202

**Published:** 2024-03-11

**Authors:** Brendan L. Murphy, François P. Gabbaï

**Affiliations:** Department of Chemistry, Texas A&M University, College Station, Texas 77843-3255, United States

## Abstract

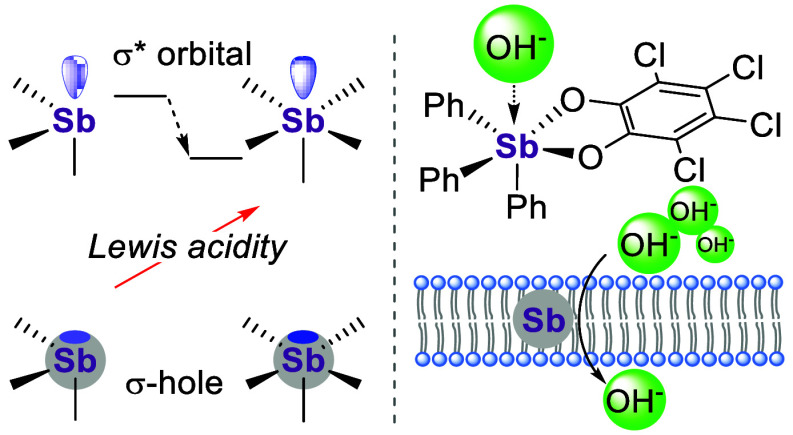

Our growing interest in the design of pnictogen-based strategies for anion transport has prompted an investigation into the properties of three simple triarylcatecholatostiboranes (**1**–**3**) of the general formula (*o*-C_6_Cl_4_O_2_)SbAr_3_ with Ar = Ph (**1**), *o*-tolyl (**2**), and *o*-xylyl (**3**) for the complexation and transport of hydroxide across phospholipid bilayers. A modified hydroxypyrene-1,3,6-trisulfonic acid (HPTS) assay carried out in artificial liposomes shows that **1** and **2** are potent hydroxide transporters while **3** is inactive. These results indicate that the steric hindrance imposed by the three *o*-xylyl groups prevents access by the hydroxide anion to the antimony center. Supporting this interpretation, **1** and **2** quickly react with TBAOH·30 H_2_O ([TBA]^+^ = [^*n*^Bu_4_N]^+^) to form the corresponding hydroxoantimonate salts [^*n*^Bu_4_N][**1**-OH] and [^*n*^Bu_4_N][**2**-OH], whereas **3** resists hydroxide coordination and remains unperturbed. Moreover, the hydroxide transport activities of **1** and **2** are correlated to the +V oxidation state of the antimony atom as the parent trivalent stibines show no hydroxide transport activity.

The transport of anions across membranes requires water-stable and appropriately lipophilic compounds capable of capturing an anion before shuttling it through the membrane.^1^ Research in this field, which has implications for new treatments for diseases, has typically been dominated by hydrogen bond donors.^2^ Recently, this field has witnessed the entry of main group derivatives, whose Lewis acidic properties can be leveraged for the transport of anions across phospholipid bilayers.^3^ This possibility has been unambiguously established for simple antimony(V) derivatives^4^ such as the stibonium cation [Ph_4_Sb]^+^ (**A**, Figure 1), which complexes and transports halides across phospholipid bilayers.^5^ Interestingly, efforts from the past few years have also shown that appropriately substituted antimony(III) derivatives, such as stibine **B**, function as chloride anion transporters.^3b,6^ The ability of such stibines to transport anions derives from the pnictogen bond (PnB) donicity of the antimony atom, which can engage the chloride anion *via* a σ hole interaction, probably dominated by electrostatic forces.^7^ Given our interest in anion transporters that could respond to changes in the redox environment of the medium,^3c,5b,8^ we have now decided to establish whether the anion transport properties of stibines could be turned on *via* oxidation of the antimony center.

**Figure 1:**
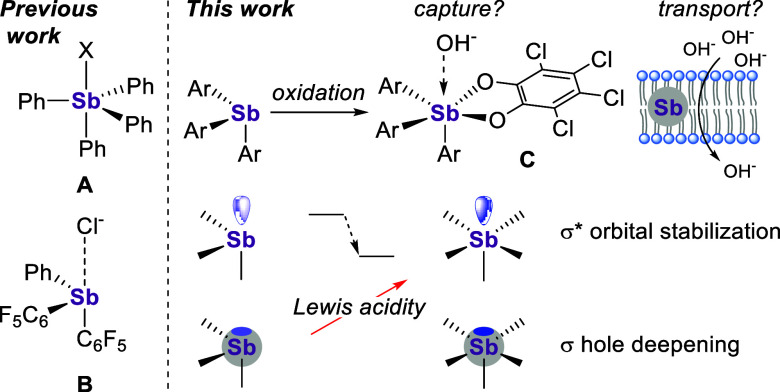
Important precedents and investigative framework of this study.

As part of our ongoing efforts toward the development of transporters for hard anions, we chose to test this idea with hydroxide as the anionic cargo. This choice was guided by the relevance of hydroxide transport to pH gradient modulation across biological interfaces.^9^ We also note that strategies for selective hydroxide transport have implications beyond biology, with applications in new strategies for alkaline nuclear tank waste treatment.^10^ However, the targeted and reversible complexation of the hydroxide anion in aqueous solutions is complicated by the high hydration energy of this anion (−430 kJ/mol)^11^ and its tendency to decompose its receptors. These difficulties have been explicitly overcome only in a few documented cases,^2c,12^ adding urgency to the present work.

While σ hole interactions in neutral triarylstibines might in principle enable hydroxide transport, we contend that the stronger PnB donor properties of neutral antimony(V) derivatives (or stiboranes) would deliver greater activities.^13^ Thus, we turned our attention to a subclass of stiboranes called catecholatostiboranes (**C**, Figure 1),^14^ which can complex hard anions^4a^ in competitive media.^15^ The properties of these derivatives can be easily adjusted by the choice of a catecholate ligand^15,16^ and its aryl substituents,^13^ providing several avenues for tuning their anion complexation properties. Importantly, even with their high Lewis acidity, catecholatostiboranes are generally air and water stable,^15,17^ features that we have exploited for the biphasic capture of fluoride.^16^ Although their hydroxide complexation behavior has not been structurally documented, the isolation of stiborane–water adducts,^17a,18^ reversible acid–base titration assays,^17b^ and similar complexation chemistry of fluoride and hydroxide suggest their aptitude in this regard. With these precedents as a backdrop, we set out to investigate the hydroxide transport properties of catecholatostiboranes and compare them to those of their trivalent precursors.

Compounds **1**–**3** were synthesized *via* treatment of their parent stibines^19^ with one equivalent of *o*-chloranil in CH_2_Cl_2_ (Figure 2). While **1** is a known compound,^4a,17b,17d^ yellow-colored **2** and **3** are new and have thus been fully characterized (Figures S1–S4). The ^1^H NMR spectra of **2** and **3** reveal four and two aromatic resonances, respectively, indicating fluxional structures with equivalent rings at room temperature. Solutions of **1** and **2** in coordinating solvents (e.g., DMSO, THF, etc.) lose their typical yellow color over time, a feature that we have previously ascribed to the coordination of a solvent molecule at the σ*(Sb–C) orbital.^17c,20^ Nevertheless, these associations are benign to the receptors, as **1** and **2** are stable in solutions of *d*_6_-DMSO/D_2_O (9.5:0.5 (v/v)) (Figures S9 and S10).

**Figure 2:**
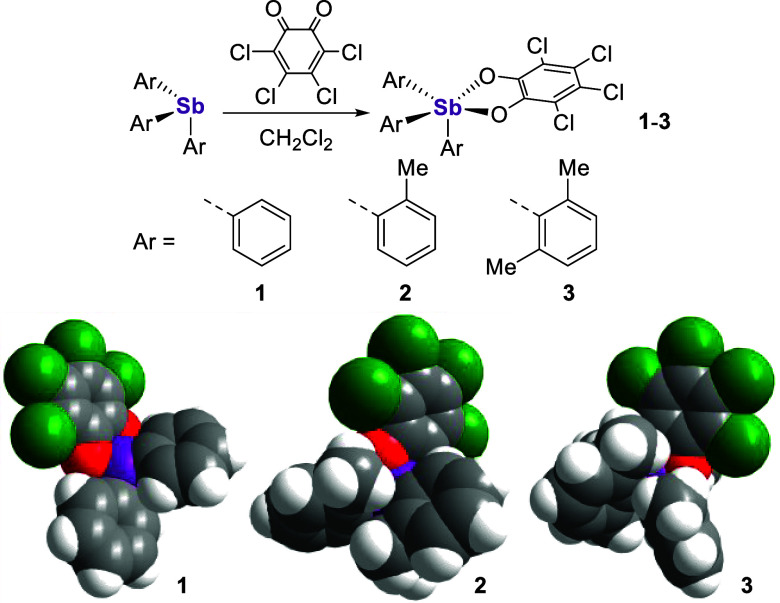
Top: Synthesis of stiboranes **1**–**3**. Bottom: Space-filling models of the solid-state structures of stiboranes **1**(17d)–**3**. For **3**, only one of the structures found in the asymmetric unit is shown. Color code: purple (Sb), red (O), green (Cl), gray (C), white (H).

In the solid state, **2** adopts a geometry best described as distorted square pyramidal, as evidenced by its crystallographically determined τ_5_ value^21^ of 0.04, which is assigned to the one-sidedness of the *o*-tolyl substituents. Interestingly, the bulkier derivative **3** adopts a less distorted trigonal bipyramidal geometry as indicated by its τ_5_ value of 0.61, which is very close to that determined for **1** (0.65).^17d^ The variation seen in the τ_5_ values of these simple derivatives illustrates the molecular flexibility of these pentavalent derivatives.^22^ Because crystal structures only incompletely capture the possible geometries that these compounds can adopt, the value above should not be overinterpreted with regard to the accessibility of the antimony center. A more pertinent parameter that captures this feature is the percent volume buried (%*V*_bur_) of the antimony center of these derivatives, which stands at 84.0%, 91.3%, and 96.3% for **1**, **2**, and **3**, respectively. This can be seen when visualizing the crystal structures of the compounds as space-filling models (Figure 2), whose antimony atoms are increasingly shielded from view with increasing substitution on their aryl rings. Accordingly, this effect accompanies an increase in the lipophilicities of the structures, as readily captured by the computed octanol/water partition coefficient values of 7.48, 7.66, and 8.58 for **1**, **2**, and **3**, respectively.

Evidence for hydroxide anion complexation came following treatment of the corresponding Lewis acids with TBAOH·30 H_2_O in CH_2_Cl_2_/MeOH. Beginning with **1**, the characteristic yellow color of the stiborane faded away quickly when treated with TBAOH·30 H_2_O, and the corresponding hydroxoantimonate [**1**-OH]^−^ was isolated as a [^*n*^Bu_4_N]^+^ salt (Figure 3). The resulting colorless solid has been characterized by multinuclear NMR as well as X-ray crystallography (Figures S5 and S6). Inspection of the solid-state structure confirms the presence of a hydroxide anion bound to the antimony atom, which adopts a distorted octahedral geometry like the previously characterized fluoride analog [**1**-F]^−^.^17b^ The Sb–O_1_ distance in [**1**-OH]^−^ of 2.0191(16) Å is below the sum of the covalent radii of the two elements (2.05 Å)^23^ with a formal shortness ratio of 0.98,^24^ confirming a strong PnB. Indeed, this bond length is on par with the Sb–O bond length found for Ph_4_Sb–OH (2.048 Å)^25^ as well as a methoxide-bound stiborane (2.0381(10) Å).^26^

**Figure 3:**
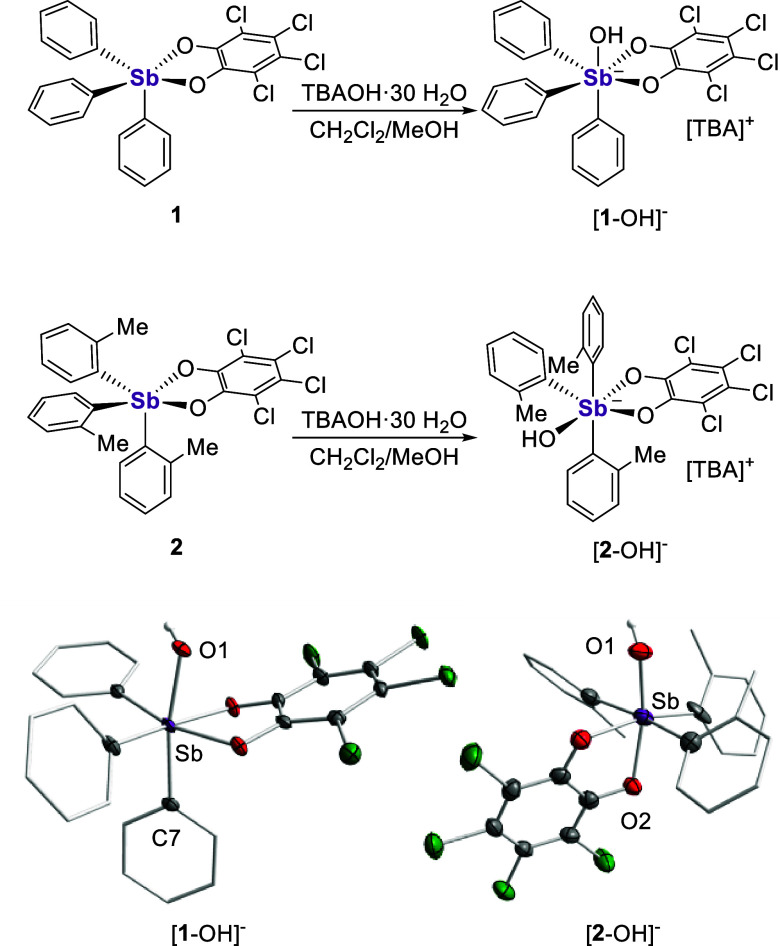
Top: Syntheses of [^*n*^Bu_4_N][**1**-OH] and [^*n*^Bu_4_N][**2**-OH]. Bottom: Solid-state structures of [**1**-OH]^−^ and [**2**-OH]^−^. Hydrogen atoms (excluding the hydroxide hydrogens) and [^*n*^Bu_4_N]^+^ counterions are omitted for the sake of clarity. Selected bond lengths (Å) and angles (deg) for [**1**-OH]^−^: Sb–O_1_ = 2.0191(16) and O_1_–Sb–C_7_ = 169.96(8). Selected bond lengths (Å) and angles (deg) for [**2**-OH]^−^: Sb–O_1_ = 2.006(4) and O_1_–Sb–O_2_ = 165.29(15).

Despite its crowded surface, **2** readily reacts with TBAOH·30 H_2_O, furnishing crystals of [^*n*^Bu_4_N][**2**-OH] that reveal the docking of hydroxide to the antimony atom (Figure 3). While it also adopts a distorted octahedral geometry, [**2**-OH]^−^ positions its bound hydroxide *trans* to its oxygen ligand at a Sb–O_1_ distance of 2.006(4) Å. Such a configuration, which has some precedence with other bulky stiborane Lewis adducts,^17c^ further speaks to the flexibility of Sb(V) species.^22b^ No hydroxide-bound adduct of **3** could be obtained and no reaction was seen following the addition of excess TBAOH·30 H_2_O to **3** in CDCl_3_/*d*_6_-DMSO (1:1 (v/v)) by ^1^H NMR (Figure S11), suggesting that the steric bulk around the antimony atom prohibits contact with the anion.^27^

Electrostatic potential maps of the hydroxide-accepting structures of **1** and **2** were then generated by removing the bound hydroxide from [**1**-OH]^−^ and [**2**-OH]^−^, respectively. This approach allows us to identify the antimony-centered σ holes associated with *V*_S,max_ values of 46.8 and 57.6 kcal·mol^–1^, respectively, at the sites of hydroxide anion complexation (Figure 4). The depths of these σ holes are commensurate with the electron-withdrawing abilities of the element *trans* to the electropositive surface,^28^ with the more polar Sb–O bond of **2** giving rise to a deeper σ hole than the Sb–C bond of **1**. A similar analysis of the parents Ph_3_Sb and (*o*-tol)_3_Sb returned significantly shallower σ holes on the trivalent antimony atom, pointing to the role of oxidation in augmenting the Lewis acidity of the pnictogen atom.^13^ Oxidation also lowers the antimony-centered acceptor LUMO energy, which was computed at the relaxed geometries (−1.70 eV for **1**(29) and −1.59 eV for **2** vs. −0.59 eV for Ph_3_Sb and −0.50 eV for (*o*-tol)_3_Sb).

**Figure 4:**
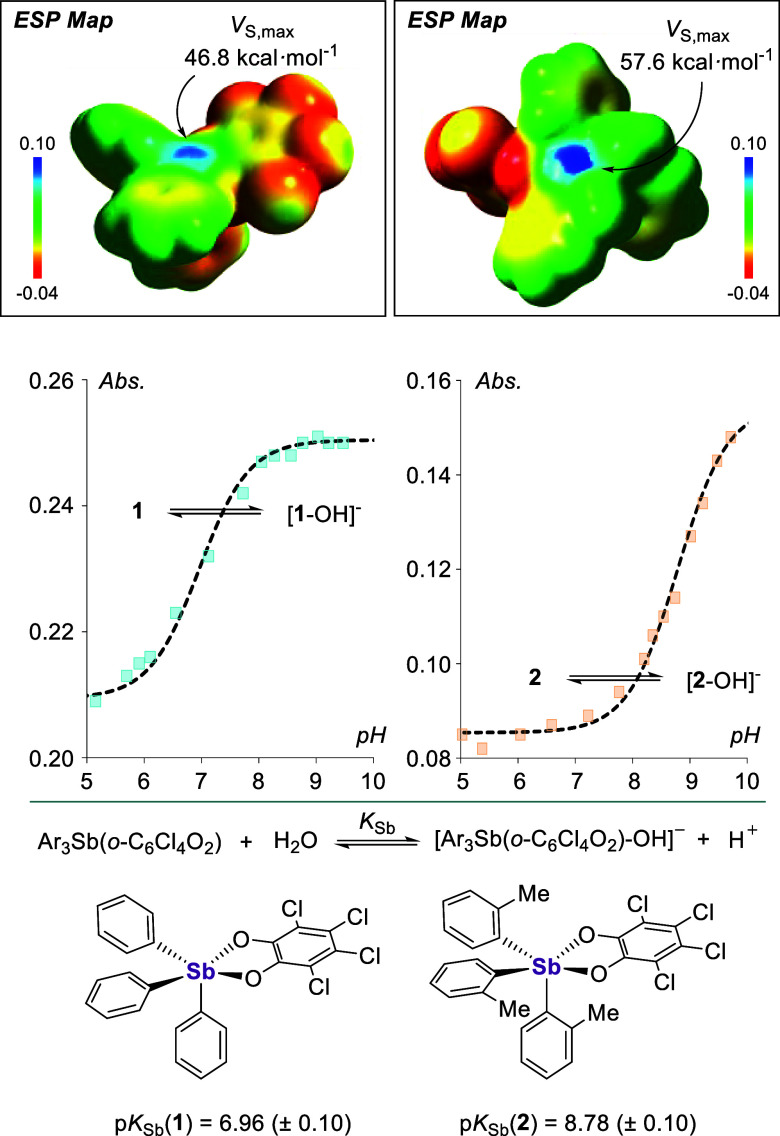
Top: Electrostatic potential maps of the hydroxide-accepting geometries of **1** and **2** (isovalue: 0.0015 au, gradient scale values given in a.u.). Middle: Spectrophotometric titration data collected for **1** and **2** in H_2_O/THF (9.5:0.5 (v/v), 0.01 M ethanolamine, 0.045 M Triton X-100) upon addition of aqueous NaOH. Bottom: Summary of p*K*_Sb_ values for compounds **1** and **2**.

Prior to testing these compounds for anion transport, it became imperative to confirm their ability to engage hydroxide anions in aqueous media. We thus sought to measure their p*K*_Sb_ values — that is, the pH values at which the stiboranes are bound by hydroxide — by acid–base titration monitored *via* UV–vis spectroscopy, as we had previously reported for **1**.^17b^ We repeated this experiment using a slightly different medium, leading to p*K*_Sb_ values of 6.96 (± 0.10) and 8.78 (± 0.10) for **1** and **2**, respectively (Figure 4). The higher value measured for **2** reflects how the *ortho*-methyl substituents lower the Lewis acidity of the antimony center. This passivating substituent effect becomes extreme in the case of **3** as indicated by the lack of spectral changes in the pH 5–10 window chosen for this study. While we propose that the formation of [**1**-OH]^−^ and [**2**-OH]^−^ results from the direct complexation of a hydroxide anion, we cannot rule out the involvement of an incipient water adduct that undergoes subsequent deprotonation.

With these results in hand, we then set about testing these compounds as hydroxide transporters using large unilamellar vesicles (LUVs) prepared with 1-palmitoyl-*sn*-2-oleoyl-glycero-3-phosphocholine (POPC) and loaded with HPTS, a fluorescent pH indicator (Figure 5).^30^ These LUVs were subjected to a KOH pulse to produce a pH gradient, followed by an injection of the potassium-cation-selective transporter valinomycin.^2c^ Hydroxide transport from the external medium to the vesicle interior was then initiated by the addition of the antimony derivative, and HPTS fluorescence was monitored (see Section 4.2 in the Supporting Information for more details). To begin, the injection of a tetrahydrofuran (THF) blank revealed no significant hydroxide transport. However, addition **1** and **2** as THF solutions elicited rapid and potent dissipation of the pH gradient (Figure 5).

**Figure 5:**
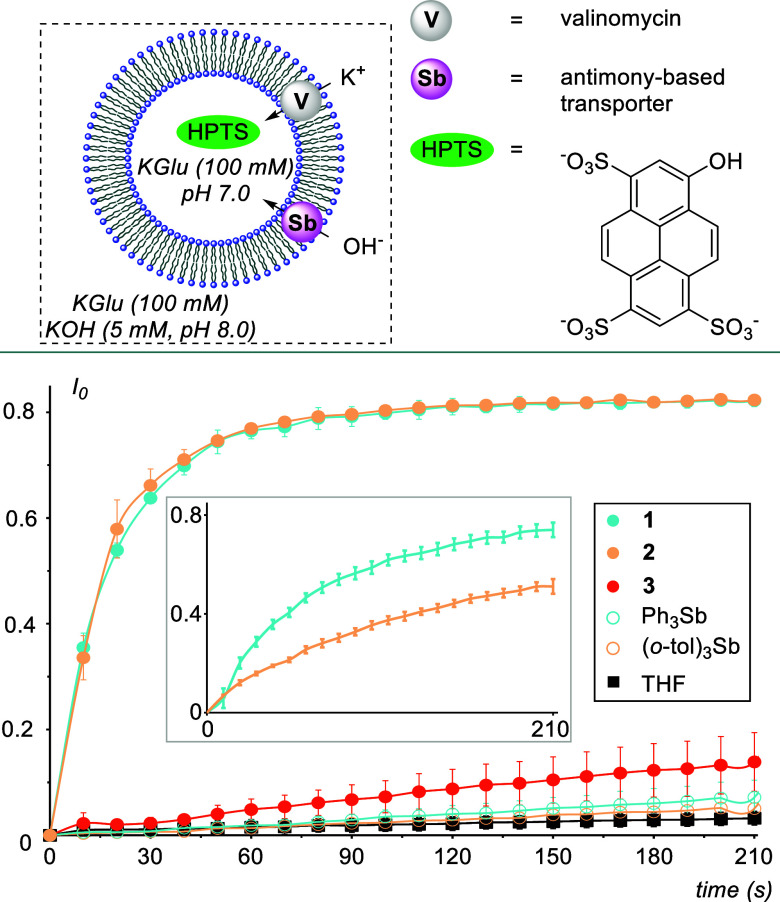
Top: Experimental design for hydroxide transport facilitated by the various antimony compounds in the presence of valinomycin (0.005 mol% with respect to lipid concentration). Bottom: Valinomycin-coupled hydroxide influx into POPC vesicles triggered by addition of a THF solution of stiboranes **1**–**3**, Ph_3_Sb, or (*o*-tol)_3_Sb (2 mol% with respect to lipid concentration) as monitored by HPTS fluorescence. POPC concentration: 0.1 mM. Error bars represent the standard deviations of three experiments. Inset shows the superior hydroxide transport activity of **1** compared to **2** at 0.05 mol%, near the EC_50_ value of **2**.

Further analysis reveals that **1** outperforms **2** in this regard as indicated by the EC_50_ values at 210 s of 6.9 × 10^–3^ mol% for **1** and 37 × 10^–3^ mol% for **2** (Figures S15 and S16). We ascribe this differential activity to the diminished Lewis acidity of **2** compared to that of **1** due to the steric and electronic effects of the appended *ortho*-methyl groups. This notion is further supported by the lack of activity of **3**, whose Lewis acidic surface is inaccessible to the target anion. Further mechanistic insight can be gleaned from the derived Hill coefficients of each active stiborane being close to 1, indicating the transport of one hydroxide anion per one receptor. Satisfyingly, administering **2** to POPC LUVs loaded with carboxyfluorescein revealed no leakage, indicating that its activity likely does not result from the destabilization of the vesicle membrane (Figure S17). We then became curious whether their corresponding stibines of **1** and **2** were capable of hydroxide transport as well. Neither Ph_3_Sb nor (*o*-tol)_3_Sb was active as a hydroxide transporter at 2 mol% with respect to lipid concentration, suggesting that the enhanced PnB donor properties of the Sb(V) center are necessary for hydroxide transport.

These results serve to introduce new members of a growing class of hydroxide transporters based on strong but reversible antimony-centered PnBs of neutral catecholatostiboranes. These compounds can be leveraged to adjust transmembrane pH gradients *via* the transport of hydroxide. This activity is controllable on two fronts. First, the superior PnB donor properties of the +V oxidation state provide catecholatostiboranes with the ability to shuttle hard anions across phospholipid membranes when compared to their lower valent counterparts. Second, the hydroxide transport activity of these stiboranes can be tuned by prohibiting or enabling access to the antimony(V) surface. Investigation into the transport of other anions by this class of transporters is underway in our laboratory.

## Supporting Information Available

Experimental and computational details (PDF)
