# Supplementary material for: Tunable Pnictogen Bonding at the Service of Hydroxide Transport across Phospholipid Bilayers
Source: J Am Chem Soc. 2024 Mar 11;146(11):7146–51. doi: 10.1021/jacs.4c00202 (PMC10958499; doi:10.1021/jacs.4c00202)
Supplement: Supplementary file 1 — ja4c00202_si_001.pdf [file ja4c00202_si_001.pdf]

Tunable pnictogen bonding at the service of hydroxide transport across phospholipid bilayers

Brendan L. Murphy and François P. Gabbaï

Department of Chemistry, Texas A&M University, College Station, Texas 77843-3255, United States

Supporting Information

This PDF file includes

Contents

1. Synthesis and characterization
- **1.1. General considerations**
- **1.2. Synthetic procedures & NMR spectra**

2. Computational details
- **2.1. Methods**
- **2.2. Optimized geometries and coordinates of the antimony compounds**
- **2.3. Percent volume buried (%V_{Bur})**
- **2.4. Electrostatic potential map (ESP) and sigma hole ($V_{S,max}$)**
- **2.5. Computed partition coefficients (logK_{ow})**

3. Experimental hydroxide affinity measurements monitored by UV-vis spectroscopy

4. Hydroxide transport studies
- **4.1. Preparation of vesicles**
- **4.2. Hydroxide transport in the presence of valinomycin**
- **4.3. Hill plot measurements and analysis**
- **4.4. 5(6)-carboxyfluorescein (CF) non-specific leakage assay**

5. References

1. Synthesis and characterization

1.1. General considerations

1,[1] tris(*o*-tolyl)stibine ((*o*-tol)$_3$Sb),[2] and tris(2,6-dimethylphenyl)stibine ((*o*-xylyl)$_3$Sb)[3] were prepared according to literature procedures with spectra that matched those reported. The following materials were purchased from their associated suppliers and used without further purification:

- Acros: Tetrabutylammonium hydroxide 30-hydrate (TBAOH·30 H_2O); 3,4,5,6-tetrachloro-1,2-benzoquinone (*o*-chloranil)
- Amresco: Triton X-100
- Alfa-Aesar: Ethanolamine
- Avanti Polar Lipids: Mini-extruder
- BioWorld: Valinomycin
- GE Healthcare – Life Sciences: Sephadex G-50
- Sigma Aldrich: 1-Palmitoyl-2-oleoyl-sn-glycerol-3-phosphocholine (POPC)
- TCI America: 8-Hydroxypyrene-1,3,6-trisulfonic acid (HPTS); potassium gluconate (KGlu)

Solvents used were ACS reagent grade and used as received. ^{1}H and ^{13}C spectra were recorded at room temperature on a Bruker Avance 500 spectrometer. Chemical shifts are given in ppm and are referenced to residual ^{1}H or ^{13}C solvent peaks. Elemental analyses (EA) were performed at Atlantic Microlab (Norcross, GA). Spectrophotometric acid-base titrations were performed using a Shimadzu UV-2502PC UV-Vis spectrometer and a ThermoScientific Orion pH SJ Gel Semi-Micro pH probe attached to a Corning pH/Ion Analyzer 350. Fluorimetry assays were performed with a 75 W xenon lamp integrated in a PTI QuantaMaster 40 fluorescence spectrophotometer.

All crystallographic measurements were performed at 110(1) K using a Bruker D8 QUEST diffractometer (graphite monochromated Mo-Kα radiation, λ = 0.71073 Å). In each case, a specimen of suitable size and quality was selected and mounted onto a nylon loop. Integrated intensity information for each reflection was obtained by reducing the data frames using APEX3.[4] The semi-empirical method SADABS was used for the absorption corrections.[5] The structures were solved by direct methods using ShelXT[6] and refined against F^2 with anisotropic temperature-dependent parameters for all non-hydrogen atoms using ShelXL[7] using Olex2.[8] All H-atoms were geometrically placed and refined using a riding model. Diamond4 was used for final data presentation. The structural data has been deposited with the Cambridge Structural Database. Positional disorder was found to affect one of the *o*-tolyl rings of [**2**-OH][^{n}Bu$_4$N]. This disorder was modeled, leading to an improvement in the quality of the refinement. The structure discussed in the main text is the major component.

1.2. Synthetic procedures

Synthesis of **2**: To a solution of (*o*-tol)$_3$Sb (0.188 g, 0.48 mmol) in CH_2Cl_2 (1 mL) was added *o*-chloranil (0.118 g, 0.48 mmol) in one portion. The resulting solution quickly changed from a deep red to a yellow color indicating the oxidation of the antimony center. This solution was allowed to stir for 5 min, after which hexanes (~5 mL) was added to afford a yellow precipitate. This precipitate was collected by filtration and washed with hexanes (3 x 10 mL). Yield: 0.205 g (67%, 0.32 mmol). Single crystals suitable for X-ray crystallography were obtained as yellow blocks *via* evaporation of a CH_2Cl_2 solution of the compound. **^{1}H NMR** (500 MHz, $CDCl_3$): δ 7.50-7.48 (dd, J = 7.5 Hz & 1.0 Hz, 3H, Sb*Ar*), 7.44-7.41 (td, J = 7.5 Hz & 1.0 Hz, 3H, Sb*Ar*), 7.34-7.33 (d, J = 7.6 Hz, 3H, Sb*Ar*), 7.29-7.26 (t, J = 7.6 Hz, 3H, Sb*Ar*), 2.39 (s, SbAr-C*H$_3$*). **^{13}C NMR** (126 MHz, $CDCl_3$): 144.92 (s), 142.88 (s), 139.43 (s), 133.64 (s), 132.14 (s), 131.65 (s), 126.81 (s), 120.56 (s), 116.44 (s), 23.98 (s). Elemental analysis calculated for $C_{27}H_{21}Cl_4O_2Sb$: C 50.59, H 3.30; found: C 50.40, H 3.23.

Synthesis of **3**: To a solution of (*o*-xylyl)$_3$Sb (0.300 g, 0.69 mmol) in CH_2Cl_2 (1 mL) was added *o*-chloranil (0.168 g, 0.69 mmol) in one portion. The resulting solution quickly changed from a deep red to a yellow color indicating the oxidation of the antimony center. This solution was allowed to stir for 5 min, after which hexanes (~5 mL) was added to afford a yellow precipitate. This precipitate was collected by filtration and washed with hexanes (3 x 10 mL). Yield: 0.389 g (83%, 0.57 mmol). Single crystals suitable for X-ray crystallography were obtained as yellow blocks *via* evaporation of a CH_2Cl_2 solution of the compound. **^{1}H NMR** (500 MHz, $CDCl_3$): δ 7.25-7.22 (t, J = 7.4 Hz, 3H, Sb*Ar*), 7.09-7.07 (broad d, J = 7.4 Hz, 6H, Sb*Ar*), 2.47 (s, 18H, SbAr-C*H$_3$*). **^{13}C NMR** (126 MHz, $CDCl_3$): 147.56 (broad s), 144.55 (s), 141.81 (broad s), 130.57 (s), 129.77 (s), 119.90 (s), 116.74 (s), 24.17 (s). Elemental analysis calculated for $C_{30}H_{27}Cl_4O_2Sb$: C 52.75, H 3.98; found: C 52.61, H 3.96.

Synthesis of [**1**-OH][^{n}Bu$_4$N]: A solution of **1** (100.0 mg, 0.17 mmol) in CH_2Cl_2 (2 mL) was stirred at room temperature, to which a solution of TBAOH·30 H_2O (133.3 mg, 0.17 mmol) dissolved in MeOH (1 mL) was added (MeOH was chosen in this case to facilitate rapid dissolution of TBAOH·30 H_2O). The resulting solution was stirred for 15 min, and then syringe filtered through a 0.45 µm PTFE membrane. After a layer of pentane (~15 mL) was added on top, clear colorless crystals of [**1**-OH][^{n}Bu$_4$N] formed over a period of 2 h which were collected by filtration. Yield: 28.3 mg (19%, 3.3 x 10^{-2} mmol). **^{1}H NMR** (500 MHz, $CDCl_3$): δ 7.91-7.89 (m, 4H, equatorial Sb*Ar*), 7.38-7.36 (m, 2H, axial Sb*Ar$_3$*), 7.32-7.30 (m, 6H, equatorial Sb*Ar$_3$*), 7.13-7.08 (m, 4H, axial Sb*Ar$_3$*), 2.85-2.81 (m, 8H, ^{n}Bu$_4$N), 1.36-1.33 (m, 8H, ^{n}Bu$_4$N), 1.20-1.16 (m, 8H, ^{n}Bu$_4$N), 0.91-0.88 (t, J = 7.4 Hz, 12H, ^{n}Bu$_4$N). **^{13}C NMR** (126 MHz, $CDCl_3$): 151.09 (s), 149.57 (s), 135.25 (s), 134.13 (s), 133.83 (s), 128.23 (s), 127.72 (s), 127.51 (s), 116.37 (s), 115.26 (s) 58.35 (s), 23.89 (s), 19.58 (s), 13.67 (s). Elemental analysis calculated for $C_{40}H_{51}Cl_4NO_3Sb$: C 55.97, H 6.11, N 1.63; found: C 55.71, H 5.92, N 1.49.

Synthesis of [**2**-OH][^{n}Bu$_4$N]: A solution of **2** (100.0 mg, 0.16 mmol) in CH_2Cl_2 (1.5 mL) was stirred at room temperature, to which a solution of TBAOH·30 H_2O (125.0 mg, 0.16 mmol) dissolved in MeOH (1 mL) was added (MeOH was chosen in this case to facilitate rapid dissolution of TBAOH·30 H_2O). The resulting solution stirred for 15 min, and then syringe filtered through a

0.45 μm PTFE membrane. After a layer of pentane (~15 mL) was added on top, clear colorless crystals of [**2**-OH][^{n}Bu$_4$N] formed over a period of 2 h which were collected by filtration. Yield: 86.0 mg (60%, 9.6 x 10^{-2} mmol). **^{1}H NMR** (500 MHz, d_6-DMSO): δ 7.52-7.50 (broad s, 1H, Sb*Ar*), 7.40-7.39 (d, *J* = 7.5 Hz, 2 H, Sb*Ar*), 7.12-7.09 (t, *J* = 7.0 Hz, 1H, Sb*Ar*), 7.00-6.92 (m, 4H, Sb*Ar*), 6.85-6.81 (m, 4H, Sb*Ar*), 3.18-3.14 (m, 8H, ^{n}Bu$_4$N), 2.11 (s, 9H, SbAr-CH_3), 1.59-1.55 (m, 8H, ^{n}Bu$_4$N), 1.33-1.29 (m, 8H, ^{n}Bu$_4$N), 0.95-0.92 (t, *J* = 7.3 Hz, 12H, ^{n}Bu$_4$N). **^{13}C NMR** (126 MHz, d_6-DMSO): δ 156.30 (s), 153.39 (s), 151.58 (s), 150.24 (s), 141.95 (s), 141.36 (s), 135.06 (s), 134.29 (s), 129.84 (s), 129.30 (s), 127.57 (s) 126.76 (s), 124.56 (s), 123.53 (s), 115.03 (s), 114.84 (s), 114.34 (s), 113.86, 57.53 (s), 23.04 (s), 22.27 (s), 21.64 (s), 19.17 (s), 13.43 (s). Elemental analysis calculated for $C_{43}H_{58}Cl_4NO_3Sb$: C 57.35, H 6.49, N 1.56; found: C 57.30, H 6.56, N 1.67.

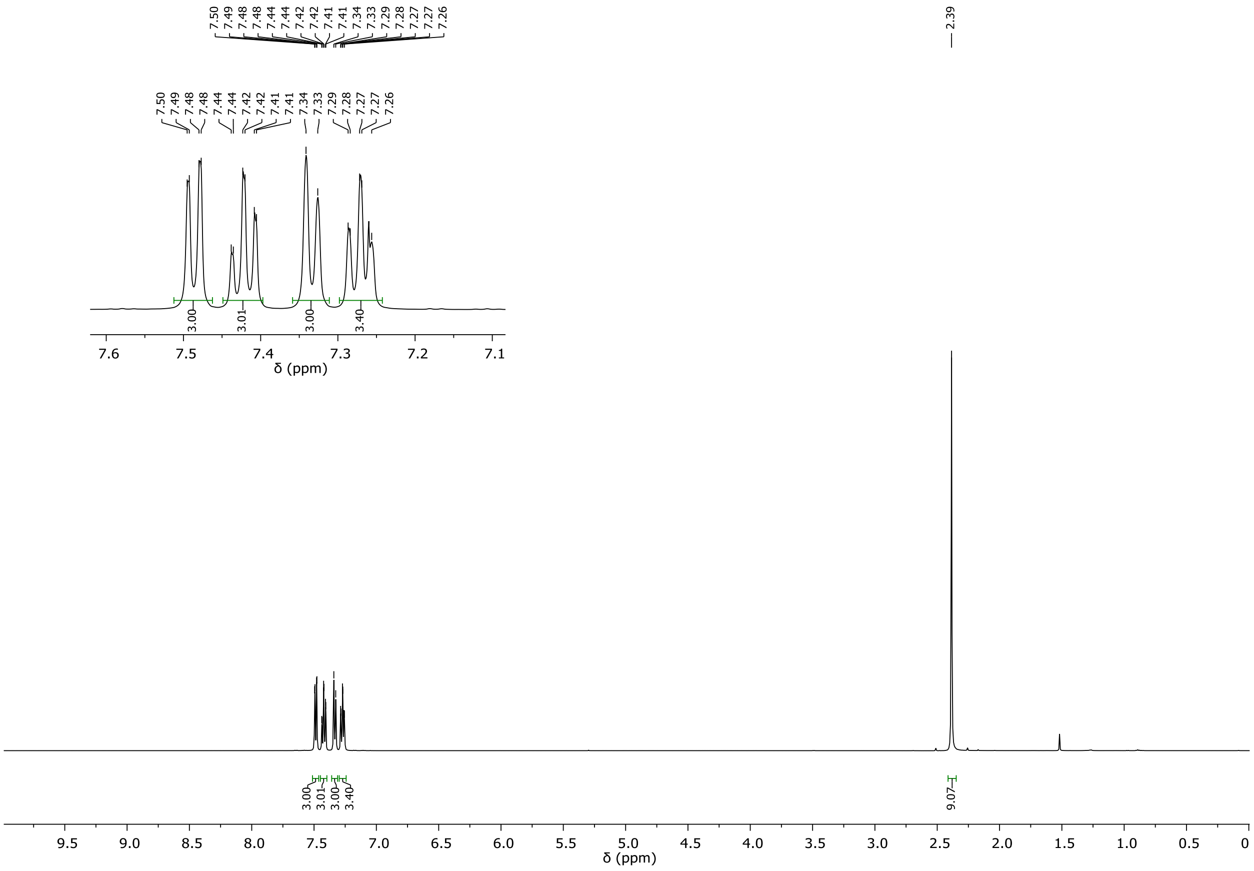

Figure S1. ^{1}H NMR ($CDCl_3$, 500 MHz) spectrum of **2**.

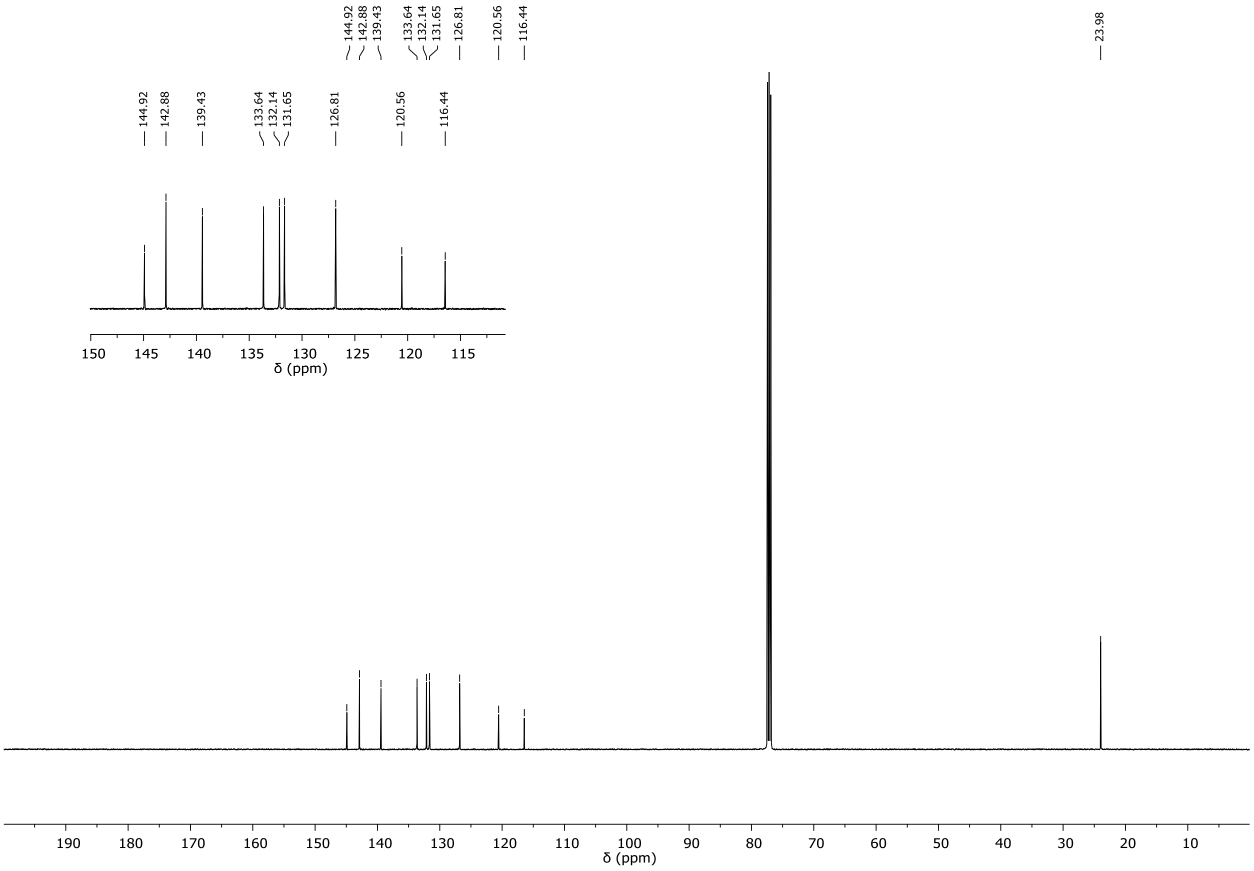

Figure S2. ^{13}C NMR ($CDCl_3$, 126 MHz) spectrum of **2**.

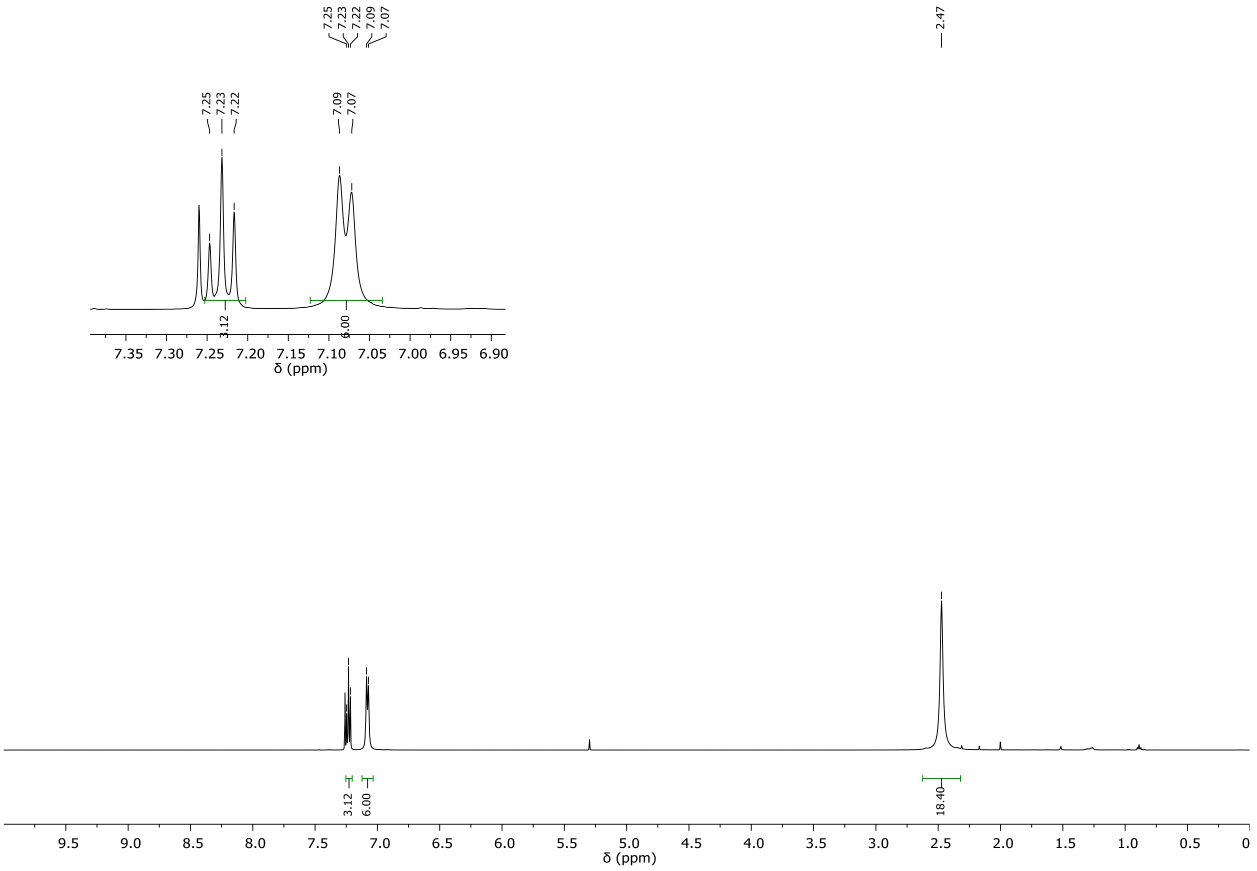

Figure S3. ^{1}H NMR ($CDCl_3$, 500 MHz) spectrum of **3**.

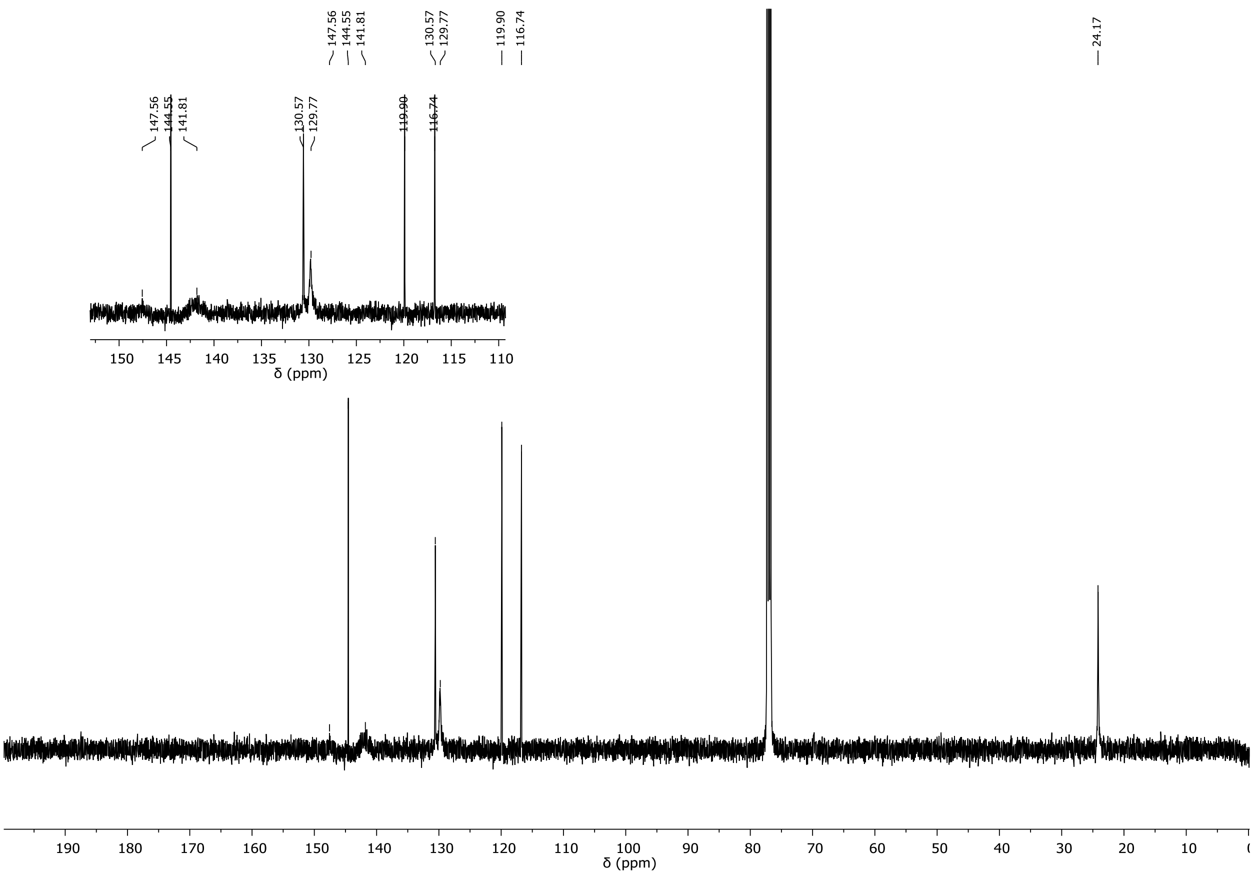

Figure S4. ^{13}C NMR ($CDCl_3$, 126 MHz) spectrum of **3**. The solvent peak is truncated for clarity.

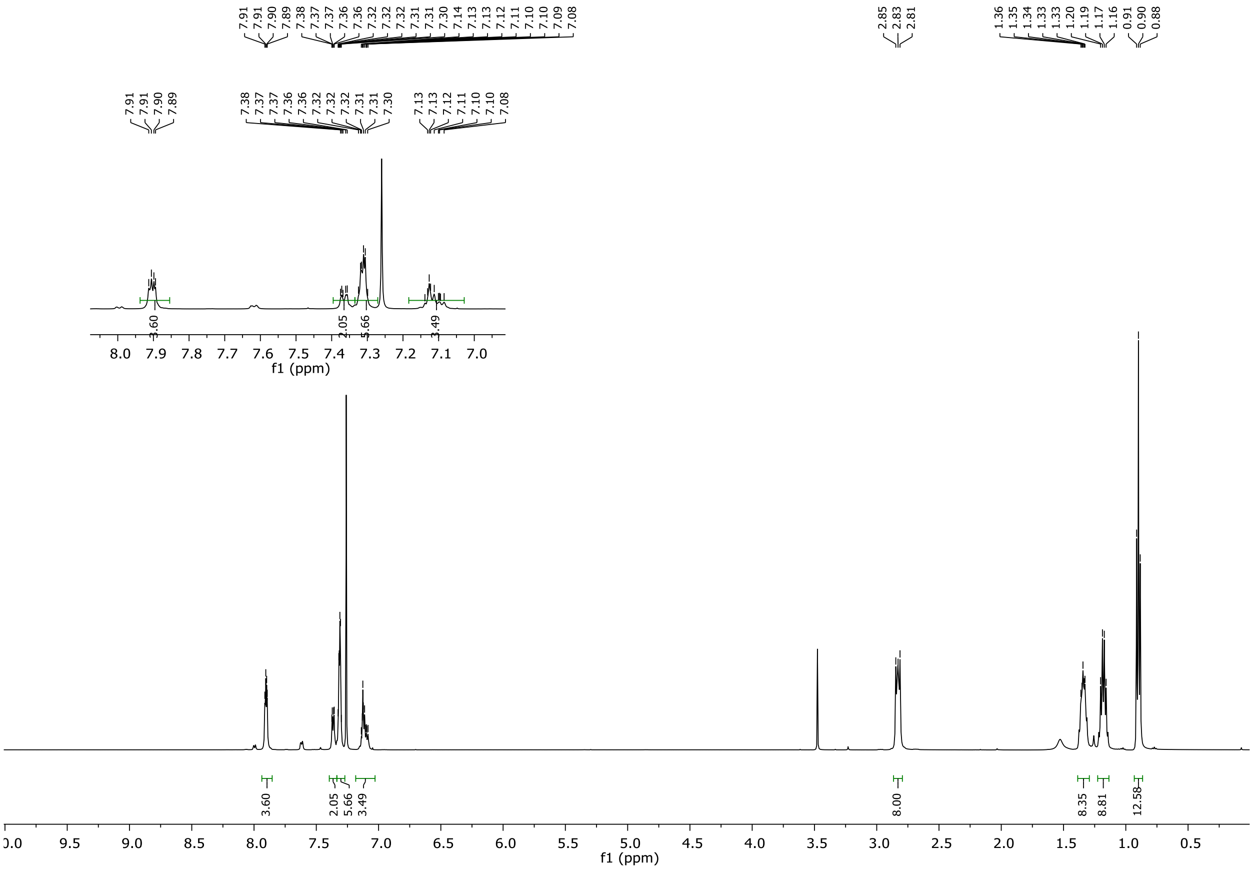

Figure S5. ^{1}H NMR ($CDCl_3$, 500 MHz) spectrum of [**1**-OH][^{n}NBu$_4$]. The peak at 3.49 ppm corresponds to methanol.

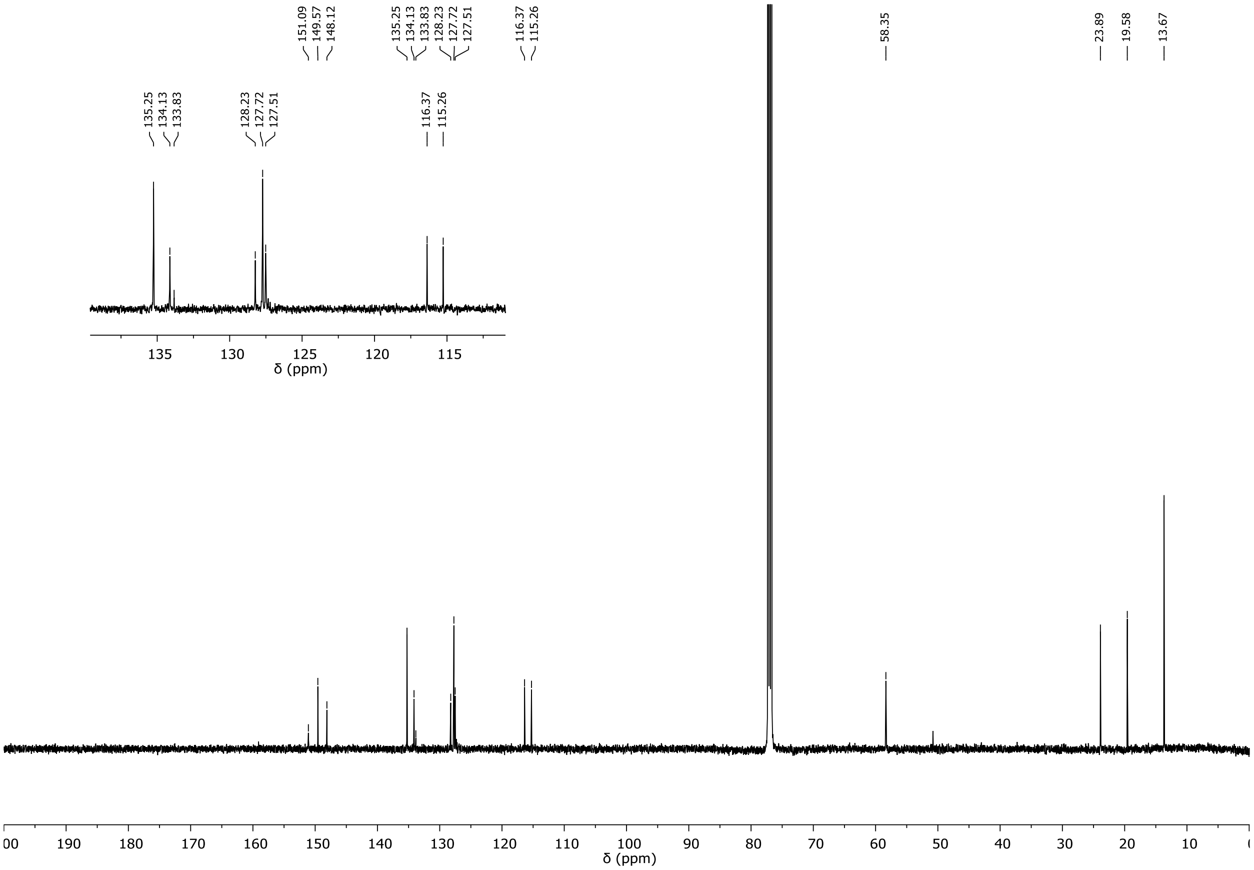

Figure S6. ^{13}C NMR ($CDCl_3$, 126 MHz) spectrum of [**1**-OH][^{n}NBu_4].

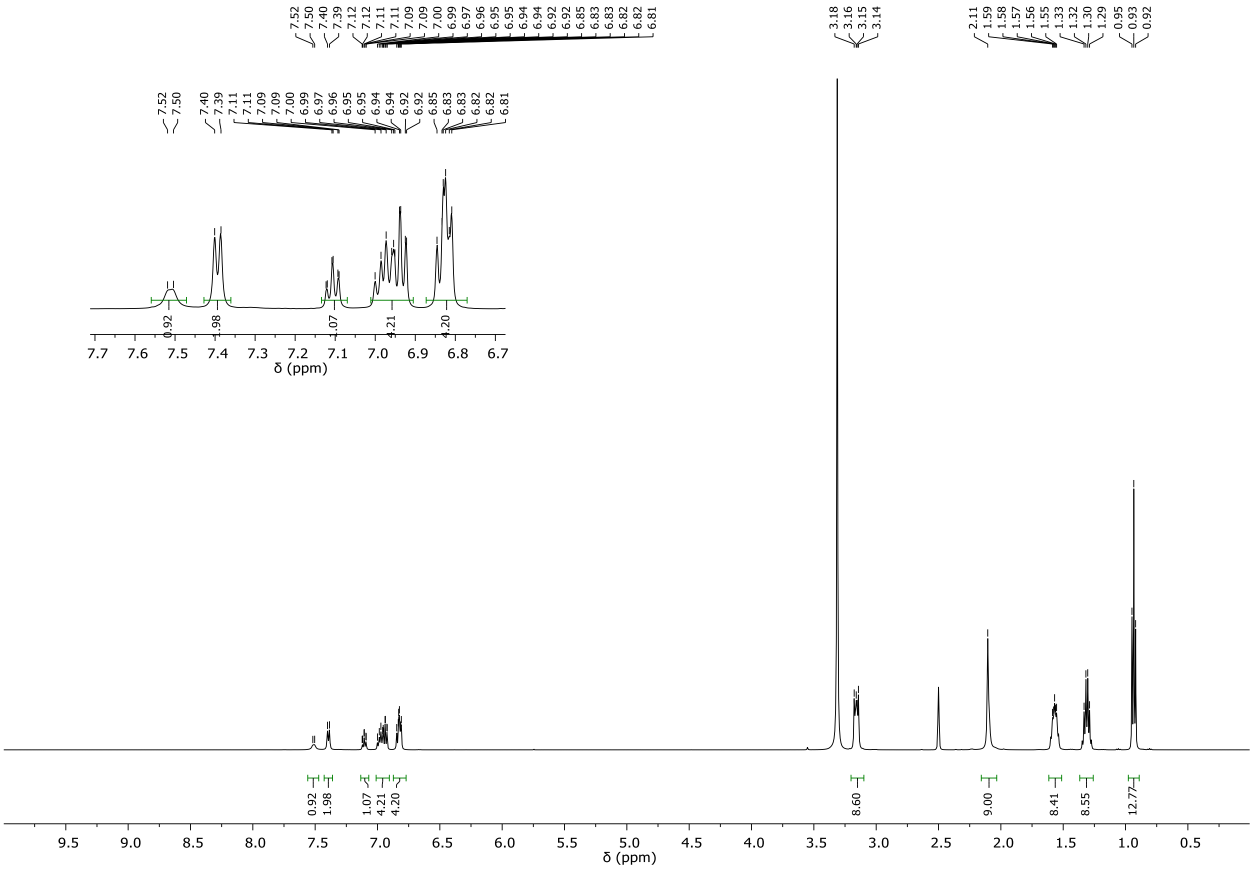

Figure S7. ^{1}H NMR (d_6-DMSO, 500 MHz) spectrum of [**2**-OH][$^{n}NBu_4$]. The peak at 3.31 ppm corresponds to water found in the solvent.

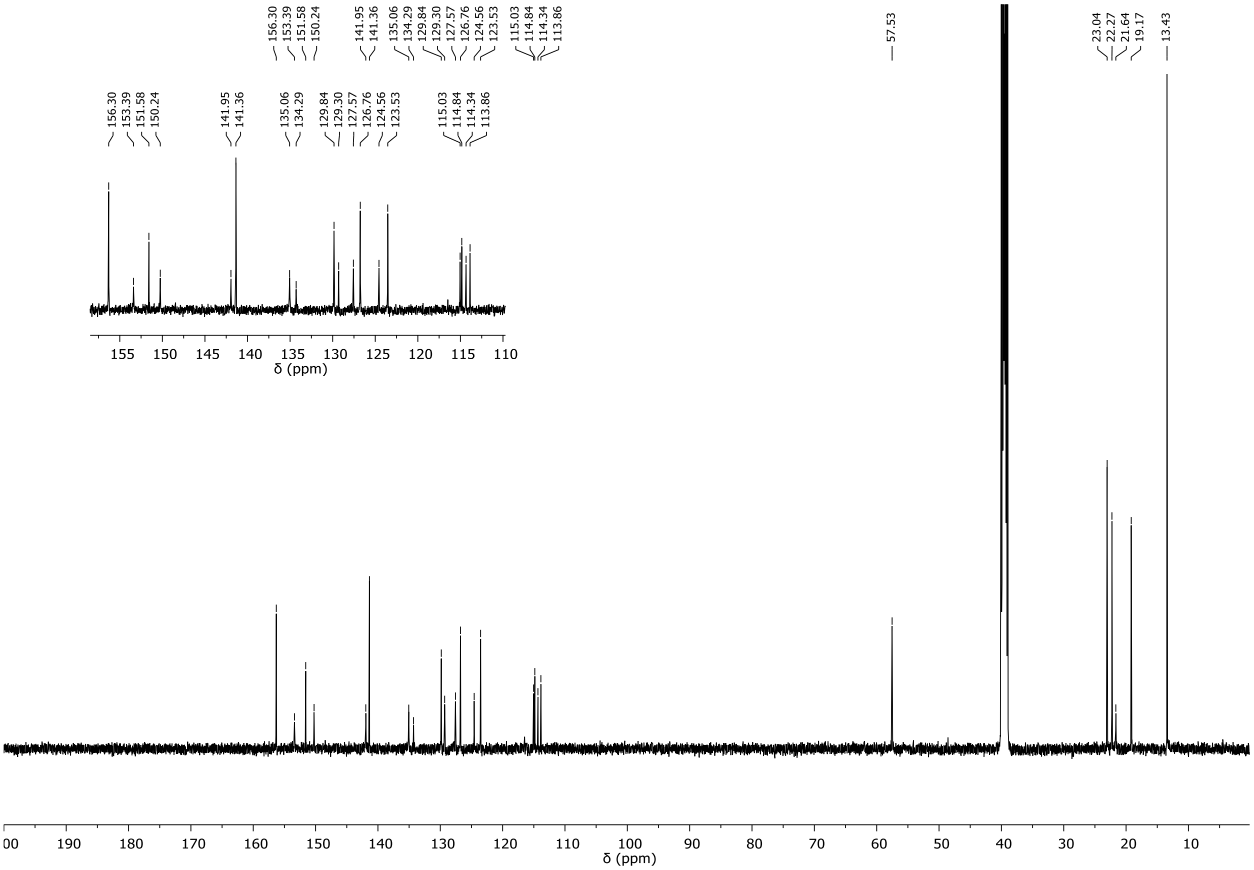

Figure S8. ^{13}C NMR (d_6-DMSO, 126 MHz) spectrum of [**2**-OH][^{n}NBu$_4$]. Solvent peak is truncated for clarity.

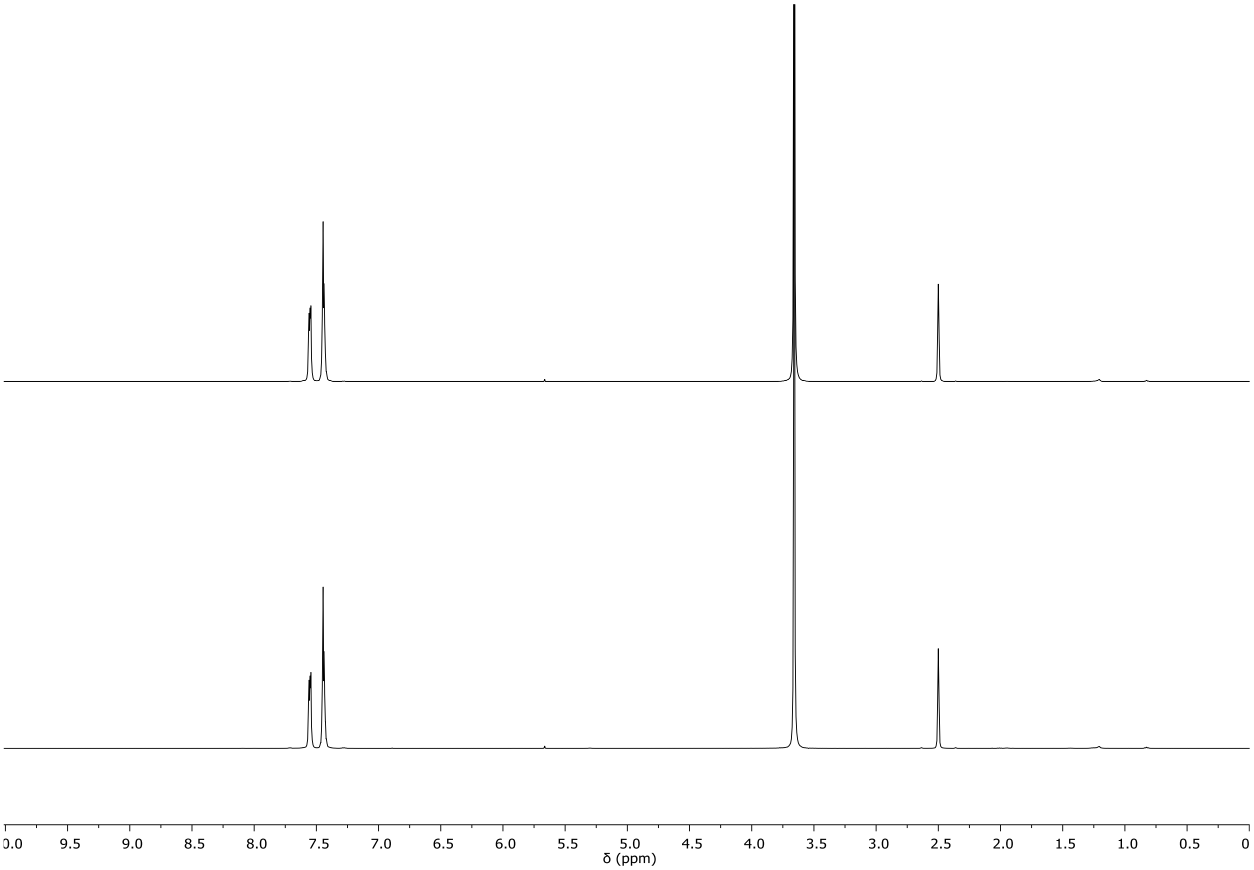

Figure S9. ^{1}H NMR (d_6-DMSO:D_2O (9.5:0.5 (v/v)), 500 MHz) of **1** at 0 h (top spectrum) and 7 h (bottom spectrum).

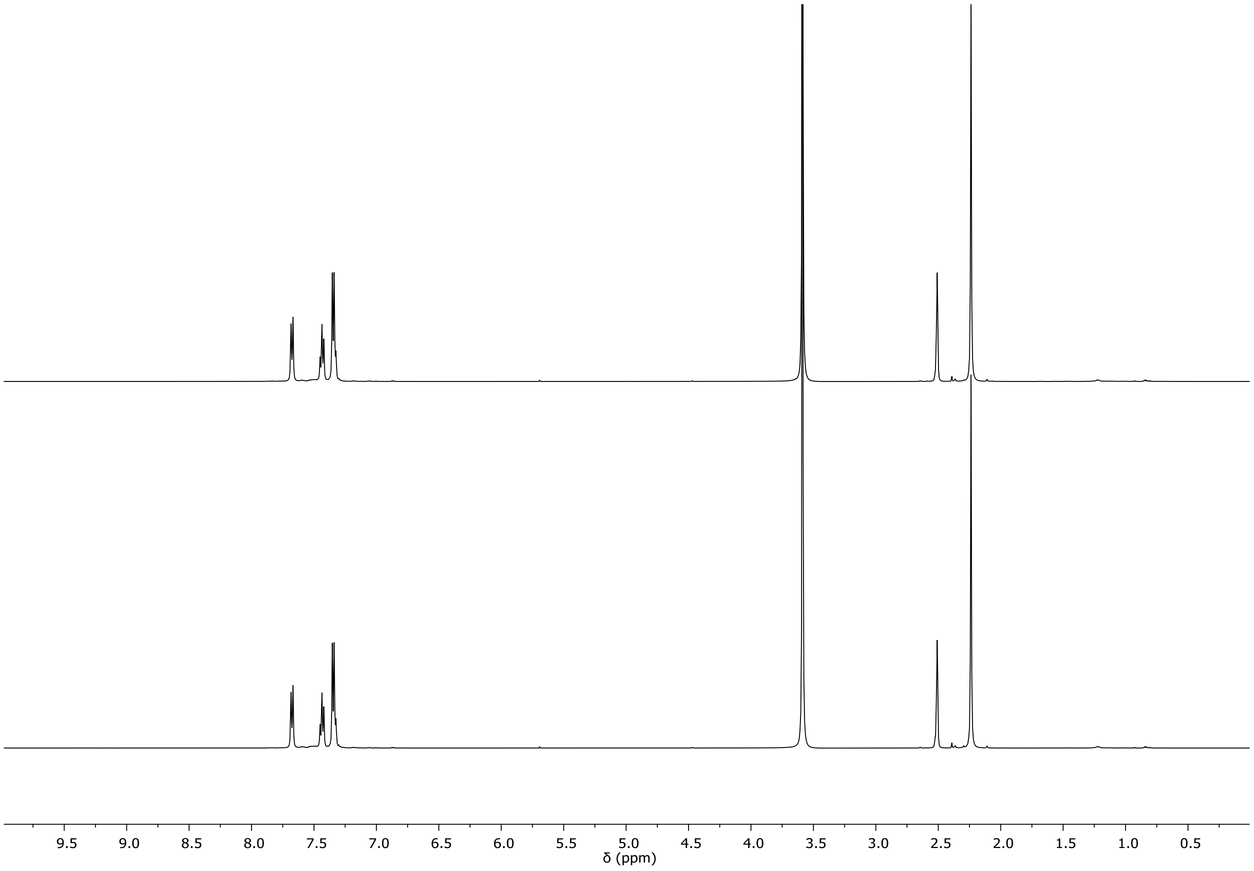

Figure S10. ^{1}H NMR (d_6-DMSO:D_2O (9.5:0.5 (v/v)), 500 MHz) of **2** at 0 h (top spectrum) and 7 h (bottom spectrum).

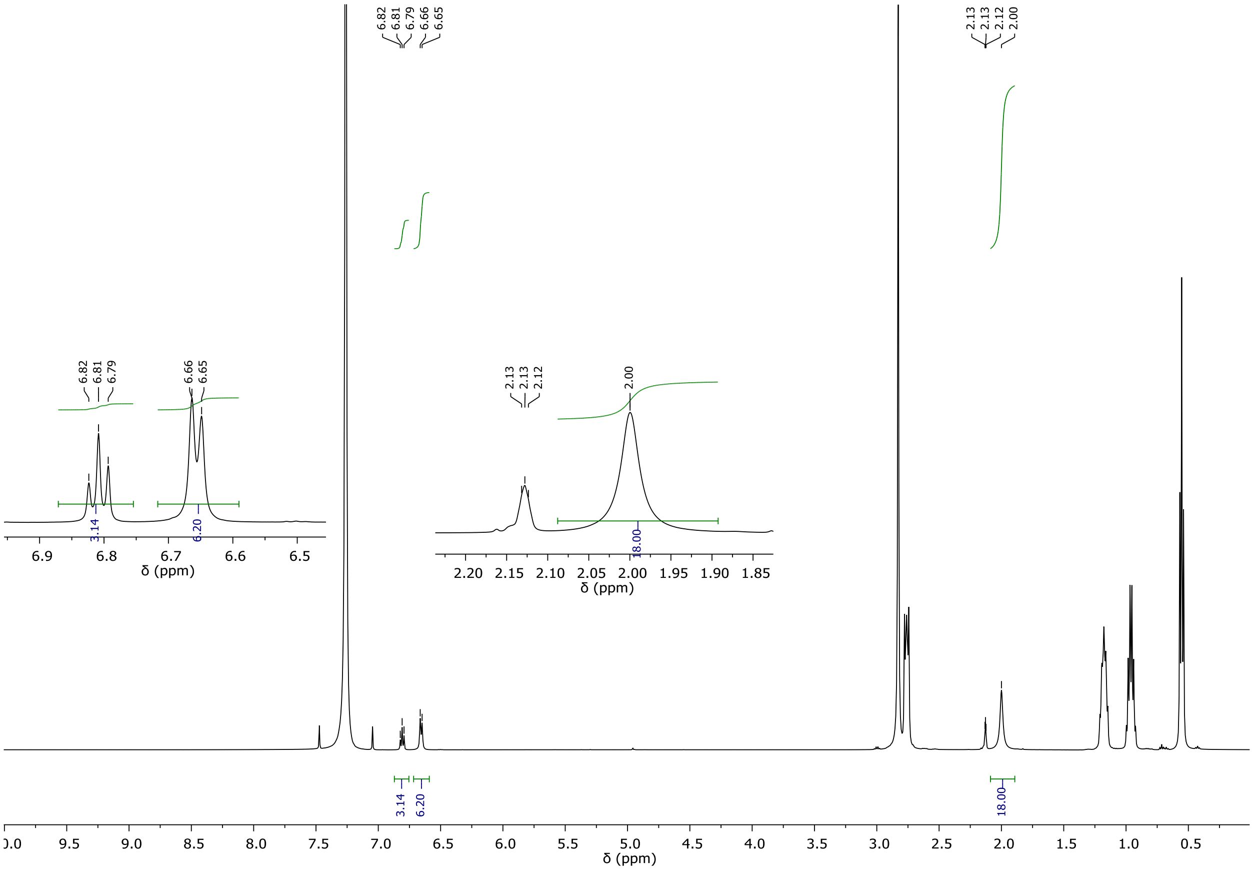

Figure S11. ^{1}H NMR ($CDCl_3$:d_6-DMSO (1:1 (v/v)), 500 MHz) spectrum showing the lack of reactivity of **3** towards excess TBAOH·30 H_2O. The spectrum is referenced to $CDCl_3$. The peak at 2.13 ppm corresponds to d_6-DMSO and the peak near 2.90 ppm corresponds to water. Because no reaction was observed with a mixture of CH_2Cl_2 and MeOH, **3** was subjected to a less competing environment of $CDCl_3$ and d_6-DMSO. The lack of desymmetrization at either the aryl protons (left inset) and alkyl protons (right inset) indicates that the antimony center has not bound the hydroxide anion.

2. Computational details

2.1. Methods

Density functional theory (DFT) structural optimizations were performed with the Gaussian 16 program.[9] In all cases, the crystal structure geometries[10] were optimized using the B3LYP functional[11] and the following mixed basis sets: aug-cc-pVTZ-PP[12] for Sb, 6-311G(d)[13] for Cl, & 6-31G[14] for C,O, and H. For all optimized structures, frequency calculations were performed in order to confirm the absence of imaginary frequencies. Single point calculations carried out at the optimized geometry with the B3LYP functional and the following mixed basis sets: aug-cc-pVTZ-pp for Sb & 6-311+g(2d,p).

2.2. Optimized geometries and coordinates of the antimony compounds

Table S1. Coordinates of the optimized geometry of **1** in the gas phase.

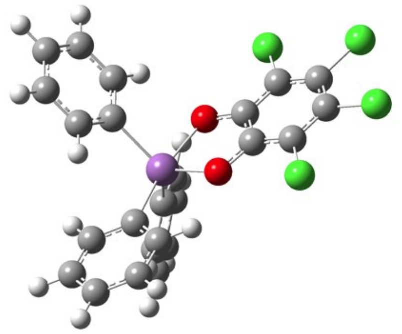

Sb	-1.23207065	-0.09447544	-0.00000020
Cl	2.40845030	3.41036702	-0.00000254
Cl	5.35131985	2.15440298	-0.00000177
Cl	5.69642581	-1.00175143	-0.00000015
Cl	3.09884341	-2.86870722	0.00000071
O	0.53163394	-1.27956537	-0.00000071
O	0.24772642	1.32096016	-0.00000204
C	1.68542500	-0.55883298	-0.00000079
C	1.54072204	0.84395002	-0.00000152
C	2.65405344	1.67728434	-0.00000172
C	3.94550949	1.10955776	-0.00000135
C	4.09674974	-0.28754643	-0.00000065
C	2.96168558	-1.12326495	-0.00000031
C	-2.63041253	1.55610729	-0.00000162
C	-4.00764806	1.25736006	-0.00000814
C	-4.95597899	2.28742855	-0.00000781
C	-4.53667457	3.62328758	-0.00000094
C	-3.17042495	3.92605934	0.00000555
C	-2.21560687	2.90018085	0.00000529
C	-1.78417660	-1.07253665	-1.82053397
C	-2.67658610	-0.44584750	-2.70785659
C	-3.03939970	-1.08558264	-3.89968164
C	-2.51915972	-2.34789874	-4.20547533
C	-1.62489823	-2.96972650	-3.32520772
C	-1.24800750	-2.33542171	-2.13627688
C	-1.78417486	-1.07253252	1.82053682
C	-1.24799334	-2.33540977	2.13629035
C	-1.62488377	-2.96971168	3.32522285
C	-2.51915766	-2.34788907	4.20548138
C	-3.03941041	-1.08558083	3.89967708
C	-2.67659690	-0.44584838	2.70785059
H	-4.34983825	0.22525812	-0.00001370
H	-6.01443229	2.04661901	-0.00001294
H	-5.27119136	4.42239082	-0.00000070
H	-2.84295089	4.96086933	0.00001080
H	-1.15962949	3.13761500	0.00001006
H	-3.08372516	0.53299334	-2.48151251
H	-3.72242724	-0.59531876	-4.58581299
H	-2.80277342	-2.84202369	-5.12918935
H	-1.21183221	-3.94375904	-3.56632476
H	-0.52656345	-2.79914618	-1.47561092
H	-0.52654055	-2.79912988	1.47563087
H	-1.21180793	-3.94373802	3.56634808
H	-2.80277120	-2.84201181	5.12919661
H	-3.72244783	-0.59532082	4.58580133
H	-3.08374565	0.53298656	2.48149847

Table S2. Coordinates of the optimized geometry of [**1**-OH]⁻ in the gas phase.

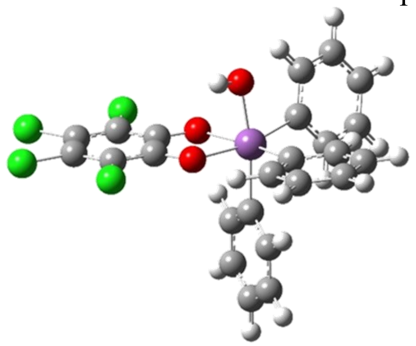

C	2.52824008	1.72421673	-0.53277492
Cl	-2.86227971	3.15905383	-0.33397798
O	0.91085359	0.03836418	-2.38482986
H	0.28272836	0.76002609	-2.59354510
Sb	1.20222106	-0.00312460	-0.37314378
C	2.01639781	3.03672552	-0.53411258
H	0.94838677	3.19024119	-0.42457344
Cl	-5.64713131	1.58380359	-0.23642709
O	-0.48441290	1.34246633	-0.35118473
C	2.87487635	4.13469308	-0.67960072
H	2.46472329	5.14136845	-0.67907942
Cl	-5.63721897	-1.59815335	-0.17504246
O	-0.47858435	-1.33345370	-0.29315641
C	4.25398363	3.94039392	-0.82617101
H	4.91765628	4.79364290	-0.93926514
Cl	-2.84482145	-3.16045522	-0.20999705
C	4.77246454	2.64042227	-0.82808844
H	5.84116135	2.47962244	-0.94568868
C	3.91494537	1.54121537	-0.68298109
H	4.33133476	0.53794771	-0.69492628
C	1.18301409	-0.05858468	1.83113597
C	1.78757073	0.96484222	2.58349368
H	2.25617418	1.80294533	2.07796490
C	1.78552528	0.92494785	3.98521000
H	2.25247555	1.72911234	4.54828213
C	1.18167835	-0.14376720	4.65665446
H	1.17819140	-0.17534156	5.74303839
C	0.57750407	-1.16995977	3.91977039
H	0.10225524	-2.00169908	4.43331839
C	0.57581933	-1.12758660	2.51940382
H	0.09726041	-1.91898602	1.95331144
C	2.56131308	-1.66754629	-0.66609051
C	2.63066082	-2.31326953	-1.91546826
H	1.99631075	-1.96244511	-2.72047446
C	3.50740353	-3.38805868	-2.10841698
H	3.54724876	-3.88317082	-3.07516568
C	4.33056134	-3.82688073	-1.06332548
H	5.01045459	-4.66092501	-1.21684540
C	4.27168132	-3.18868741	0.18033952
H	4.90497600	-3.52470234	0.99734154
C	3.38867478	-2.11756172	0.37804201
H	3.33999747	-1.64052218	1.35231230
C	-1.68265168	0.71624336	-0.30448855
C	-2.90085654	1.39914391	-0.29365018
C	-4.12388647	0.69596720	-0.25368601
C	-4.11856164	-0.70363061	-0.22769970
C	-2.89261176	-1.40220445	-0.24182467
C	-1.67587741	-0.71424119	-0.28062858

Table S3. Coordinates of the optimized geometry of **2** in the gas phase.

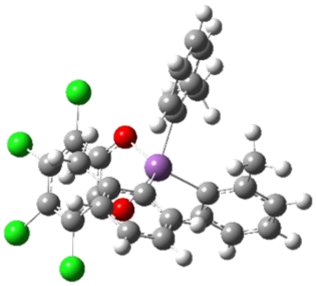

C	1.81404226	-0.65098554	0.05060033
C	3.06387565	-1.26645033	0.12860131
C	4.23297366	-0.48517798	0.02185651
C	4.14009238	0.90522265	-0.16098843
C	2.87419759	1.52313182	-0.23885989
C	1.72552767	0.74471039	-0.13274249
C	-2.32837321	1.59421315	-0.60489132
C	-2.33070174	2.84240706	0.06863202
C	-3.18785271	3.84684765	-0.41608593
H	-3.20465347	4.80606090	0.09320088
C	-4.00978038	3.64345194	-1.52961174
H	-4.65451165	4.44422400	-1.87784069
C	-3.99904974	2.41153071	-2.18631812
H	-4.63235446	2.23948630	-3.05053710
C	-3.16077467	1.39188964	-1.71878697
H	-3.16765262	0.43289316	-2.22675857
C	-1.46119628	3.14087057	1.27111357
H	-1.52965062	2.35586240	2.03379341
H	-1.76094887	4.08351291	1.73839048
H	-0.40934184	3.21684406	0.97988850
C	-1.84112177	-1.62344898	-1.30850955
C	-1.40203654	-1.72220801	-2.64857720
C	-1.91816220	-2.76772995	-3.43277361
H	-1.59415828	-2.85750017	-4.46545015
C	-2.82550296	-3.69478576	-2.90821224
H	-3.20349989	-4.49392680	-3.53769642
C	-3.23513096	-3.59756952	-1.57610283
H	-3.92698123	-4.32111036	-1.15775685
C	-2.74023476	-2.56045807	-0.77560283
H	-3.04526365	-2.50227450	0.26477717
C	-0.38016035	-0.76983636	-3.23056118
H	-0.63591644	0.28098964	-3.04472019
H	-0.29510333	-0.89857195	-4.31333197
H	0.61195208	-0.95002249	-2.79787276
C	-1.63483742	-0.53022873	2.04017995
C	-2.95061344	-0.39136908	2.55262644
C	-3.15544961	-0.68729748	3.91312410
H	-4.15910015	-0.59693618	4.31836845
C	-2.10956069	-1.09314291	4.74625528
H	-2.30547876	-1.30897983	5.79167560
C	-0.81978743	-1.23158045	4.22782930
H	-0.00178376	-1.55951792	4.86017186
C	-0.58700450	-0.95732811	2.87679871
H	0.40366788	-1.10218282	2.46585653
C	-4.14102497	0.05545084	1.72763419
H	-4.12690740	1.13542941	1.54386348
H	-5.07200354	-0.17936655	2.25221011
H	-4.18393512	-0.43397672	0.74921369
Cl	3.12716863	-2.99978716	0.35765805
Cl	5.80182019	-1.25919161	0.12065666
Cl	5.58934230	1.88082962	-0.29244441
Cl	2.70305682	3.25025915	-0.46674759
O	0.62562422	-1.31249052	0.13451611
O	0.45286455	1.25660164	-0.21132003
Sb	-1.10376464	-0.09000923	0.00451247

Table S4. Coordinates of the optimized geometry of [**2**-OH]$^{-}$ in the gas phase.

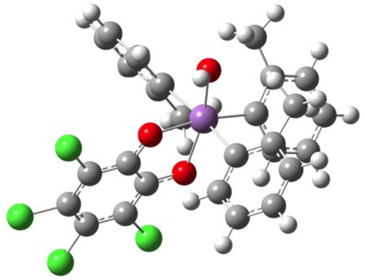

C	0.52955835	-2.04992005	-0.85745976
Cl	-2.11795446	-0.11482670	3.34572499
O	2.02691222	0.01669434	-2.40384548
H	1.63941507	0.75507298	-2.91943444
Sb	1.19925896	0.03447914	-0.54629648
O	0.18579297	-2.37583918	-2.18292234
H	0.27343587	-1.61335907	-2.94574370
Cl	-5.16407677	0.02533770	2.38016986
O	-0.15462400	0.20131511	1.10916718
C	-0.25761722	-3.65550380	-2.53271388
H	-0.52066426	-3.87540381	-3.56377313
Cl	-5.80485031	0.45453242	-0.70593238
O	-0.69570980	0.56998943	-1.48787242
C	-0.36864517	-4.63876374	-1.54587127
H	-0.71837080	-5.63676849	-1.79732495
Cl	-3.39131990	0.73721711	-2.78691547
C	-0.03628259	-4.32675743	-0.22612035
H	-0.13197951	-5.08748264	0.54551078
C	0.41492313	-3.04388350	0.14873426
C	0.76191582	-2.81752753	1.60756569
H	0.33366562	-3.61439689	2.22597756
H	0.38367999	-1.85828670	1.96734932
H	1.84721417	-2.81993631	1.76681041
C	2.90778765	-0.43016718	0.75069211
C	2.91426832	0.21923864	1.99804545
H	2.09561947	0.88454778	2.25542587
C	3.95012457	0.01588163	2.91777132
H	3.93352143	0.52520319	3.87724008
C	5.00282890	-0.84340762	2.58853650
H	5.81808869	-1.00680521	3.28851992
C	5.00563760	-1.49313669	1.35004453
H	5.82723166	-2.15866894	1.09410217
C	3.96737413	-1.30886310	0.41640475
C	4.01650670	-2.06426844	-0.89666646
H	3.73208474	-1.43012598	-1.74168478
H	3.31786844	-2.91069759	-0.89092181
H	5.02210587	-2.46176243	-1.07597238
C	1.55284511	2.22538206	-0.41429504
C	0.47367995	3.04028786	-0.02488240
H	-0.48477556	2.59048115	0.19414244
C	0.59720814	4.42963814	0.08733262
H	-0.25946995	5.02585323	0.38881466
C	1.82242897	5.03693544	-0.19622551
H	1.93607500	6.11522394	-0.12044044
C	2.90440348	4.24400203	-0.58338648
H	3.86024010	4.71316266	-0.80674283
C	2.80272453	2.84214321	-0.69555448
C	4.05400803	2.08152636	-1.09332999
H	4.78853635	2.76463145	-1.53565644
H	3.82956021	1.29034765	-1.81260412
H	4.52216103	1.60644535	-0.22285567
C	-1.74409629	0.43162611	-0.65408767
C	-3.07835042	0.49013495	-1.07409173
C	-4.13462311	0.36789847	-0.14788279
C	-3.85380970	0.17867209	1.21098373
C	-2.51260200	0.10967386	1.64501267
C	-1.45976536	0.23530882	0.73586467

Table S5. Coordinates of the optimized geometry of **3** in the gas phase.

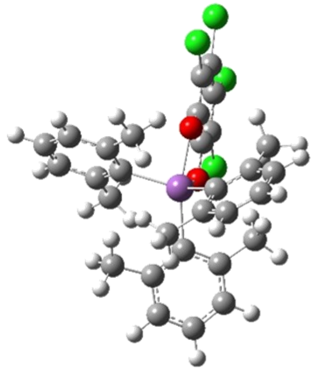

Sb	-1.04202259	-0.07476464	0.05353045
Cl	2.64568257	3.30262989	-0.65883042
Cl	3.25183223	-2.94549447	0.05215266
Cl	5.57087862	2.00864865	-0.57045680
Cl	5.87319366	-1.13161336	-0.21278474
O	0.45600544	1.26433011	-0.36625707
O	0.70527281	-1.32268284	-0.08471253
C	1.74245820	0.76761213	-0.33572994
C	-0.90653497	-0.51330213	2.17067871
C	-0.54878409	0.53254619	3.06028241
C	-2.43741027	1.62268032	-0.28330604
C	1.86595332	-0.62988300	-0.17365202
C	-0.89536954	-2.09896990	4.00396615
H	-1.00465700	-3.11692043	4.36452879
C	-3.39422924	2.06132813	0.67251811
C	-1.97187370	-1.46631171	-1.35379899
C	0.12562889	-1.53798948	-2.90201340
H	0.85347255	-2.17928164	-2.39687760
H	0.37744951	-0.50510865	-2.65115816
H	0.25693909	-1.65423808	-3.98244554
C	-0.40851265	0.22504692	4.42562778
H	-0.13997906	1.02086219	5.11376677
C	4.28286596	-0.39694964	-0.26110218
C	-0.24214407	1.95485551	2.63072162
H	0.80528797	2.04952241	2.32043349
H	-0.39836611	2.64332685	3.46690207
H	-0.84682755	2.30256977	1.79164170
C	4.15115929	0.99276864	-0.42074856
C	-3.58663650	1.48662447	2.06501475
H	-3.24843014	0.45824536	2.16934351
H	-4.64758484	1.51716264	2.33489345
H	-3.04699259	2.07613677	2.81576648
C	3.13710337	-1.20895150	-0.14020187
C	-1.03932456	-1.85184417	2.62784070
C	-3.26471960	3.31194071	-1.84099461
H	-3.21559221	3.78627197	-2.81618987
C	2.86715538	1.57699760	-0.45856923
C	-4.25369769	3.12801660	0.34003760
H	-4.98217980	3.45506450	1.07661940
C	-4.13342416	-1.36080246	0.06616760
H	-3.56579709	-1.32240843	1.00111223
H	-4.98720050	-2.02206668	0.24178948
H	-4.52503645	-0.35500152	-0.12271166
C	-2.38977714	2.24824260	-1.56454729
C	-1.97045783	-2.78737557	-3.38789754
H	-1.45460170	-3.13347469	-4.27830578
C	-3.31297897	-1.86014926	-1.10532302
C	-1.26589494	-3.03839734	1.71312301
H	-2.23569853	-3.01354364	1.20645322
H	-1.22801837	-3.96677020	2.28987638
H	-0.48820282	-3.08537314	0.94486498
C	-3.93878593	-2.74231707	-2.00178921
H	-4.95914071	-3.05553251	-1.80238664
C	-1.28988585	-1.91740916	-2.51412289
C	-0.59441060	-1.07212386	4.89876106
H	-0.48614544	-1.28686892	5.95710842
C	-3.27400087	-3.20738334	-3.13497204
H	-3.77160155	-3.88584270	-3.82044501
C	-1.45142850	1.82699136	-2.67709513
H	-0.40549657	1.93859230	-2.38092493
H	-1.61958084	2.44236253	-3.56534953
H	-1.61902881	0.78506892	-2.97220720
C	-4.18788113	3.75860830	-0.89711311
H	-4.85601473	4.58156161	-1.13034166

Table S6. Coordinates of the optimized geometry of Ph_3Sb in the gas phase.

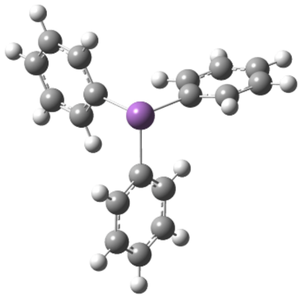

Sb	0.00034831	-0.00015366	-1.23615253
C	1.87994254	-0.13527501	-0.15756145
C	2.99800512	0.52890104	-0.70031002
C	4.24297855	0.47485363	-0.06130913
C	4.38952600	-0.25320268	1.12559605
C	3.28866571	-0.92506255	1.67026353
C	2.04136168	-0.86754663	1.03385818
C	-0.82314826	1.69533609	-0.15806085
C	-1.96065241	2.32796057	-0.69809027
C	-2.53656887	3.43310222	-0.05925631
C	-1.97630276	3.92765191	1.12473927
C	-0.84075989	3.31382599	1.66663018
C	-0.26665395	2.20487360	1.03039976
C	-1.05698023	-1.56019402	-0.15766500
C	-1.04137835	-2.86064236	-0.70017804
C	-1.71068241	-3.91149094	-0.06069387
C	-2.41385319	-3.67399034	1.12649685
C	-2.44471938	-2.38459438	1.67095655
C	-1.77126905	-1.33344080	1.03406270
H	2.90155953	1.09051048	-1.62662273
H	5.09440648	0.99425136	-0.49077612
H	5.35505098	-0.29997902	1.61968013
H	3.39895839	-1.49462447	2.58835085
H	1.19733169	-1.39697706	1.46457118
H	-2.40120297	1.96090689	-1.62218794
H	-3.41465234	3.90795517	-0.48660983
H	-2.41876885	4.78719139	1.61866469
H	-0.40028641	3.69692222	2.58242130
H	0.61630899	1.74141050	1.45901470
H	-0.50723988	-3.05821452	-1.62668484
H	-1.68707286	-4.90863711	-0.48998119
H	-2.93710689	-4.48656181	1.62094937
H	-2.99270311	-2.19505942	2.58923707
H	-1.80741167	-0.33765004	1.46448385

Table S7. Coordinates of the optimized geometry of (*o*-tol)$_3$Sb in the gas phase.

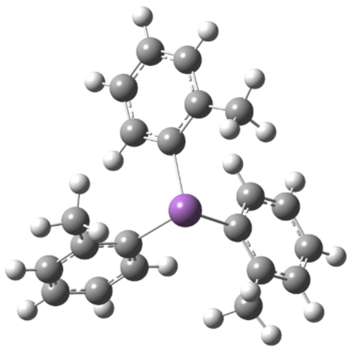

Sb	-0.00031251	-0.00002532	-1.02992228
C	0.73993444	-1.74036296	0.05429973
C	1.95223794	-2.36060632	-0.34764165
C	2.37404572	-3.52050245	0.32501839
C	1.62535153	-4.07055377	1.37095739
C	0.43232262	-3.45998819	1.76576171
C	-0.00362414	-2.30240010	1.10835059
C	2.80832734	-1.79803197	-1.46462052
C	1.13695534	1.51077916	0.05478372
C	1.06874269	2.87077437	-0.34739999
C	1.86286777	3.81567121	0.32511072
C	2.71334603	3.44200144	1.37112504
C	2.78025989	2.10356444	1.76620975
C	1.99515818	1.14756793	1.10899080
C	0.15382253	3.33126812	-1.46449616
C	-1.87707928	0.22884546	0.05529966
C	-3.02141724	-0.50849292	-0.34817387
C	-4.23645093	-0.29368540	0.32510484
C	-4.33709478	0.62752438	1.37312341
C	-3.21076307	1.35294234	1.76950458
C	-1.99062724	1.15147372	1.11152902
C	-2.96376853	-1.52875817	-1.46743539
H	-5.11368699	-0.85829972	0.02045284
H	-5.28888152	0.77509070	1.87428332
H	-3.27572493	2.06867328	2.58337766
H	-1.11911008	1.71577399	1.42748138
H	-3.95407732	-1.95177955	-1.66233576
H	-2.60093486	-1.08528495	-2.40368964
H	-2.29161336	-2.36141808	-1.22152661
H	3.67134485	-2.44248544	-1.65833820
H	2.24462478	-1.70562386	-2.40195422
H	3.19112887	-0.79918036	-1.21696556
H	3.30288346	-3.99623105	0.02156607
H	1.97374549	-4.96872052	1.87171086
H	-0.15604644	-3.87593741	2.57797301
H	-0.92942825	-1.83158972	1.42301573
H	0.28211909	4.40048447	-1.65933695
H	0.35442127	2.79563305	-2.40137753
H	-0.90280047	3.16536615	-1.21629887
H	2.04968186	0.11044551	1.42395439
H	3.43447845	1.80176161	2.57848602
H	3.31745446	4.19253198	1.87171587
H	1.81107904	4.85789962	0.0214320

2.3. Percent volume buried (%V_{Bur})

For the %V_{Bur} calculations, the crystal structure geometries of the receptors were subjected to SambVca 2.1[15] with the following parameters: the antimony atom was selected as the center of the sphere, Bondi radii was scaled by 1.17, sphere radius was set at 3.5 Å, mesh spacing for numerical integration was set at 0.10, and hydrogen atoms were included in the calculation. For the case of **3**, because there are two independent molecules in the asymmetric unit cell, both structures were subjected to this calculation and the average %V_{Bur} was taken.

2.4. Electrostatic potential maps and $V_{S,max}$ calculations

Electrostatic potential (ESP) maps were generated based on the gas-phase optimized geometries of the structures and determined at an isodensity value of 0.0015 electrons/$Bohr^3$. For the ESP maps for **1** and **2**, the bound hydroxides were removed, and the resulting structures were subjected to a single-point calculation. ESP maps were generated and analyzed using Multiwfn[16] and visualized in GaussView.[17] Multiwfn was also used to identify areas of maximum electrostatic potential ($V_{S,max}$).

2.5. Computed partition coefficients (logK_{ow})

Using a published approach[18] illustrated in Figure S12, the solute density-based implicit solvation model (SMD)[19] was used to estimate the solvation of free energy of stiboranes in water (ΔG_w) and in *n*-octanol (ΔG_o). Only the gas phase structures were optimized. The energies of the solvated molecules were obtained *via* single point calculations. The octanol-water partition coefficient K_{ow} was calculated using the equation found in Figure S12 using T = 298 K.[20] The calculation results are presented in Table S8.

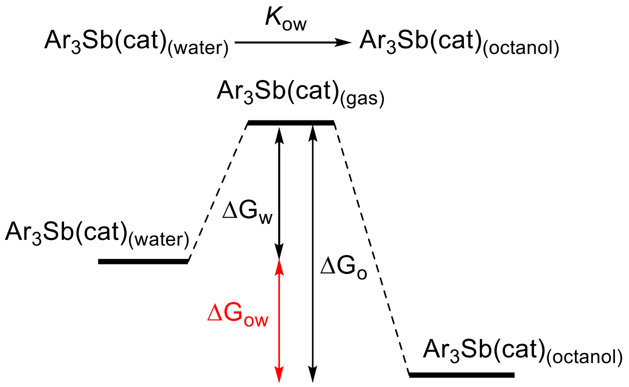

$$\log K_{ow} = \frac{-\Delta G_{ow}}{2.303RT} = \frac{\Delta G_w - \Delta G_o}{2.303RT}$$

Figure S12. Diagram representing the approach adopted to determine the partition coefficient K_{ow}.

Table S8. Computed solvation energies and octanol-water partition coefficients.

Compound	ΔG_w, Hartree (kcal/mol)	ΔG_o, Hartree (kcal/mol)	-ΔG_{ow}, kcal/mol	logK_{ow}
**1**	-0.0099327 (-6.232864684)	-0.0261793 (-16.42776228)	10.1948976	7.48
**2**	-0.0130735 (-8.230746861)	-0.0297007 (-18.63747462)	10.43372775	7.66
**3**	-0.0110569 (-6.938310985)	-0.0296893 (-18.63032101)	11.69201002	8.58

3. Experimental hydroxide affinity measurements

In a quartz cuvette was added a buffered solution (3 mL) of water:THF (9.5:0.5, 0.01 M ethanolamine, 0.045 M Triton X-100) and a small stir bar. To this solution was added a THF solution of the stiborane in study (30 µL). The solution was then titrated by incremental addition of an aqueous NaOH solution, and the resulting data were fitted to Equation 1 to yield the relevant K_{Sb}. The solutions were buffered to obtain better control of the pH near the equivalence point. After each assay, the solutions were reacidified and returned the same UV-vis trace, indicating that basification is reversible and benign.

$$Ar_3Sb(o\text{-}C_6Cl_4O_2) + H_2O \underset{}{\overset{K_{Sb}}{\rightleftharpoons}} [Ar_3Sb(o\text{-}C_6Cl_4O_2)\text{-}OH]^- + H^+$$

(Equation 1)

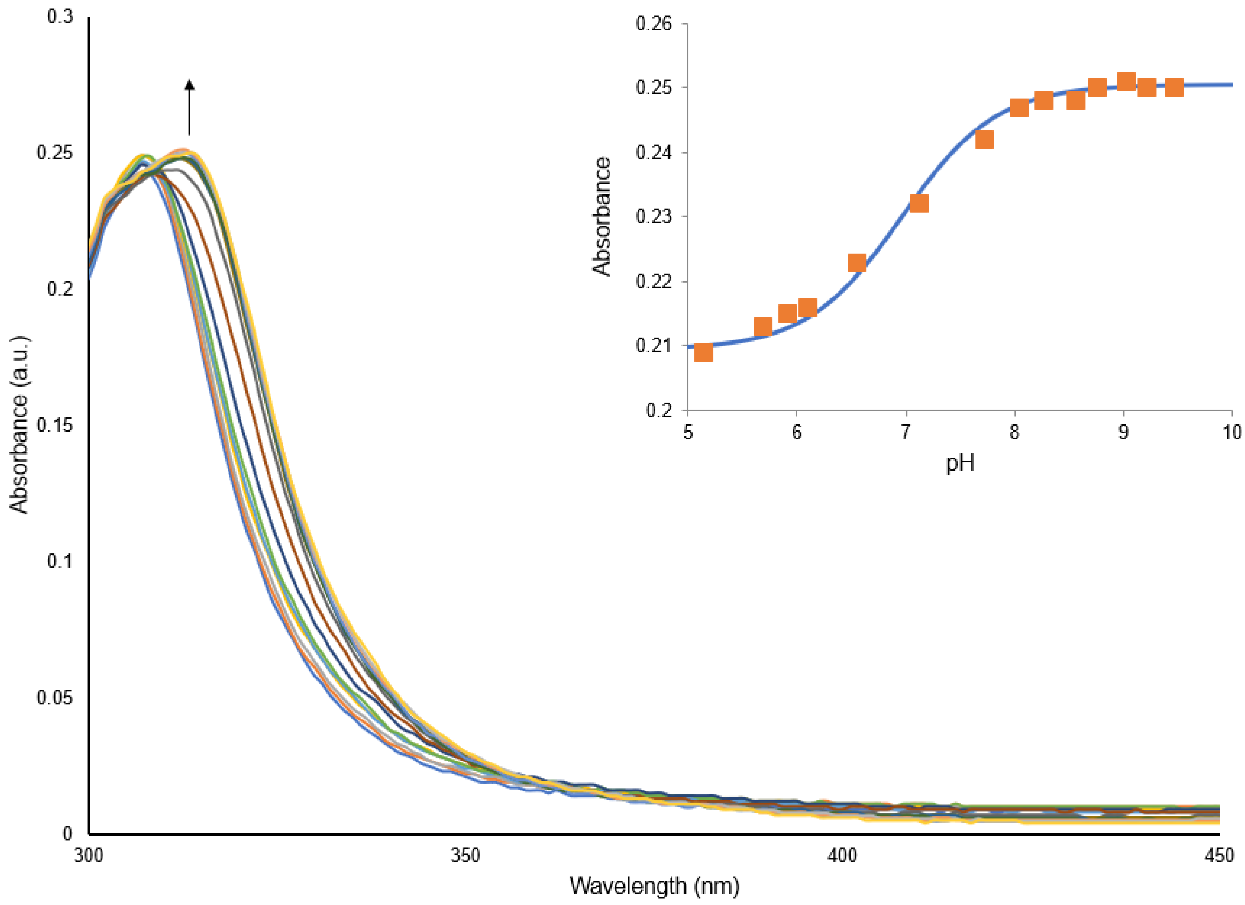

Figure S13. Spectrophotometric acid-base titration curve of **1** in water:THF (9.5:0.5 (v/v), 0.045 M Triton X-100, 0.01 M ethanolamine). The absorbance was measured at 313 nm. The resulting data were fitted to Equation 1 with ε(**1**) = 4,440 M^{-1} ε([**1**-OH]$^-$) = 5,310 M^{-1} to afford an estimated pK_{Sb} value of 6.96 (±0.10).

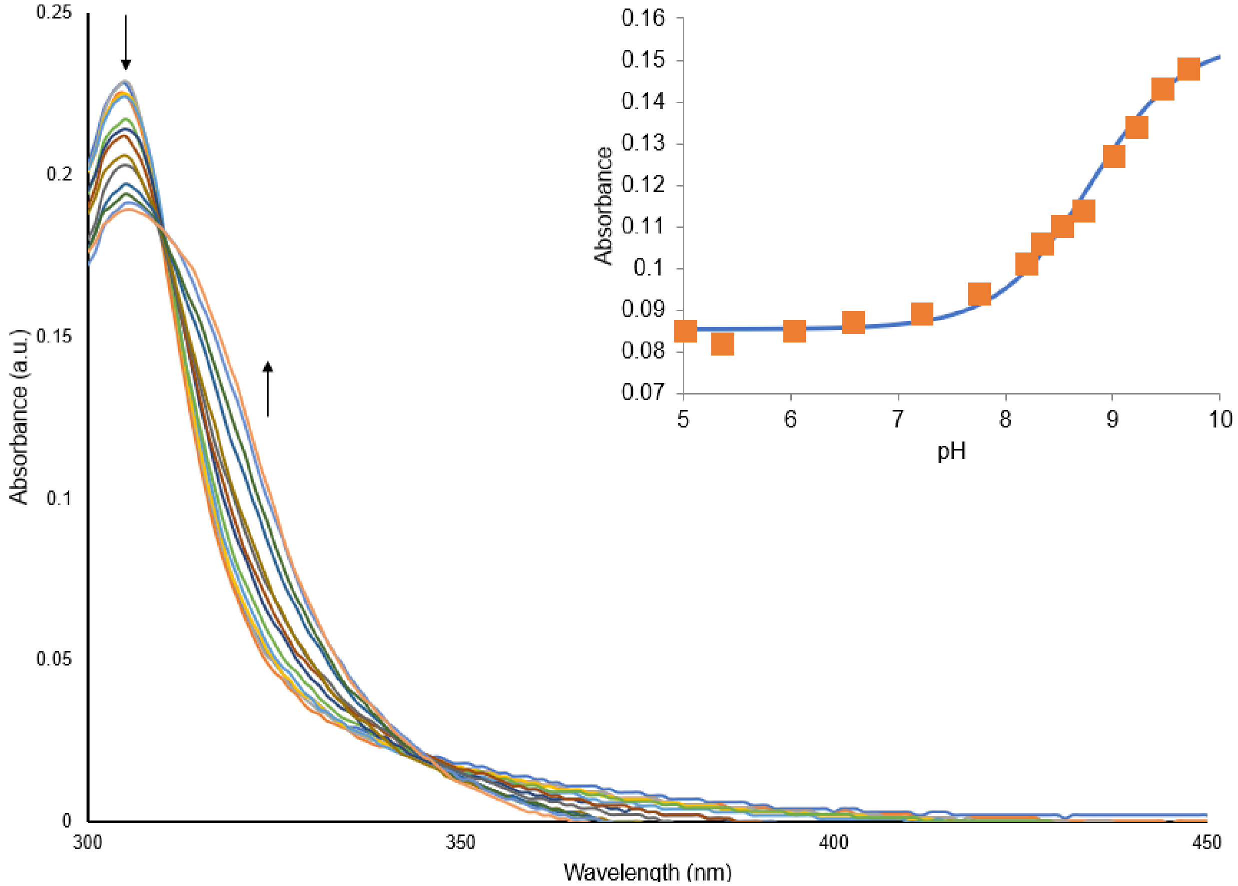

Figure S14. Spectrophotometric acid-base titration curve of **2** in water:THF (9.5:0.5 (v/v), 0.045 M Triton X-100, 0.01 M ethanolamine). The absorbance was measured at 318 nm. The resulting data were fitted to Equation 1 with ε(**2**) = 1,810 M^{-1} ε([**2**-OH]$^{-}$) = 3,280 M^{-1} to afford an estimated pK_{Sb} value of 8.78 (±0.10).

4. Hydroxide transport studies

4.1. Preparation of vesicles

4.1.1. POPC LUVs (200 nm) loaded with 8-hydroxypyrene-1,3,6-trisulfonic acid (HPTS). The vesicles were prepared according to a previously established method. A thin film of POPC lipid was prepared by evaporation of a solution of POPC (30 mg) dissolved in $CHCl_3$ (2 mL) under vacuum overnight. A buffered solution of HPTS (1 mL, 10 mM HEPES, 100 mM KGlu, pH 7.0) was then added, which was then sonicated to remove the film from the sides of the vessel. KGlu is used as an ionic strength equalizer because of its membrane impermeability. The resulting suspension was then subjected to nine freeze/thaw cycles (liquid N_2, 47 °C water bath), and extruded 33 times through a 200 nm polycarbonate membrane. To remove any extravesicular component, the vesicle suspension was eluted on a size exclusion column (Sephadex G-50) using a buffer solution (10 mM HEPES, 100 mM KGlu, pH 7.0) as the eluent. These vesicles were used within five days of synthesis, and stored at 4 °C.

4.1.2. POPC-LUVs (200 nm) loaded with CF. The vesicles were prepared similarly to Section 4.1.1., except that a buffered solution of CF (1 mL, 10 mM NaCl, 10 mM HEPES, 50 mM CF, pH 7.4) was added to the dried vesicles. The resulting suspension was then subjected to nine freeze-thaw cycles (liquid N_2, 47 °C water bath), and extruded 33 times through a 200 nm polycarbonate membrane. To remove any extravesicular component, the vesicle suspension was eluted on a size exclusion column (Sephadex G-50) using a buffer solution (10 mM HEPES, 100 mM NaCl, pH 7.4) as an eluent.

4.2. Hydroxide transport in the presence of valinomycin

The following assay was adapted from a previous report designed to determine the effect of valinomycin on the pH gradient dissipation properties of the protonophore carbonyl cyanide *m*-chlorophenyl hydrazine.[21] Vesicles containing a buffered solution of HPTS (1 mM, KGlu 100mM, HEPES 10 mM, pH 7.0) were suspended in an external buffer solution to produce a 2.5 mL solution of 0.1 mM lipid in a plastic fluorimetry cuvette that was gently stirring. The fluorescence ratio of HPTS (λ_{ex} = 460 nm, λ_{em} = 510 nm divided by λ_{ex} = 403 nm, λ_{em} = 510 nm) was monitored by fluorimetry. Addition of a base pulse of KOH (12.5 µL of a 1.0 M solution in water) generated a pH gradient that was consistently measured to be ~8.0 at a final base concentration of 5 mM, which was monitored by fluorimetry for 60 s to ensure vesicle integrity. A solution of valinomycin (2.5 µL of a 0.005 mM THF solution) was then added to the cuvette to achieve a concentration of 0.005 mol% with respect to lipid concentration and stirred for an additional 30 s to again ensure vesicle integrity. To mark the beginning of the experiment, a solution (5 µL of a 1.0 mM THF solution) of the transporter in study was then added to the cuvette to initiate transport, which was monitored for 210 s. A detergent solution (50 µL, 11% Triton X-100 in water:DMSO (7:1, v/v)) was then added to the cuvette to lyse the vesicles and destroy the pH gradient. A final reading was taken at 420 s.

The fractional fluorescence intensity (I_f) was determined using the following equation:

$$I_f = \frac{R_t - R_0}{R_{420} - R_0}$$

such that:

R_t = fluorescence ratio at time = t
R_0 = fluorescence ratio at time = 0 s (corresponding to the addition of valinomycin)
R_{420} = fluorescence ratio at t = 420 s (corresponding to the complete dissolution of the pH gradient)

4.3. Hill plot measurements and analysis

The assay described in Section 4.2 was carried out with different concentrations of stiborane (**1** or **2**). The resulting data were analyzed using Origin 2023 to generate Hill plots according to the following equation:

$$y = y_n + (y_m - y_n) \frac{x^n}{k^n + x^n}$$

such that:

x = concentration
$y = I_f$ at t = 210 s
y_m = maximum I_f value
y_n = minimum I_f value
n = Hill coefficient
k = EC_{50} value at 210 s

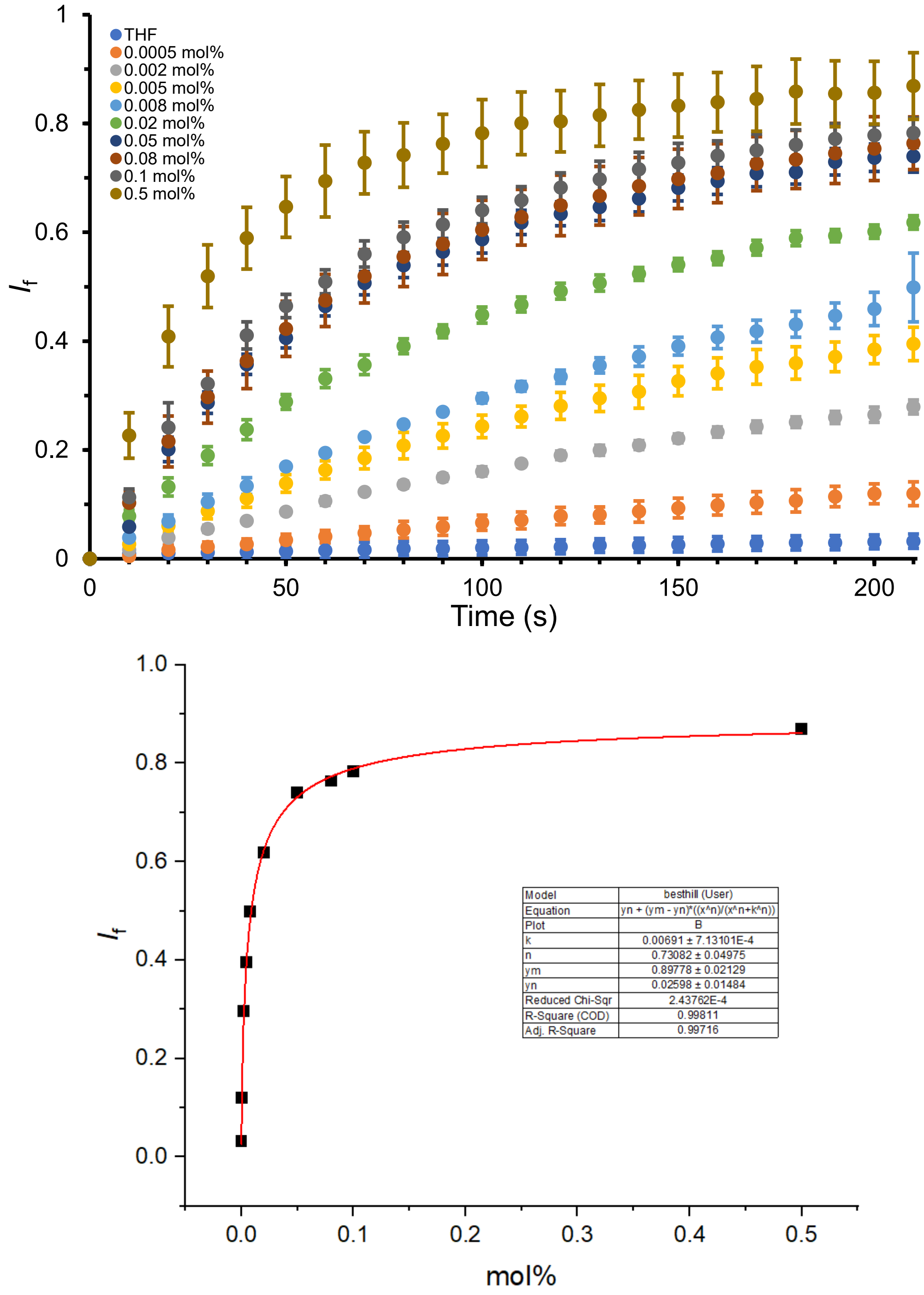

Figure S15. Hill plot analysis of the hydroxide influx into POPC vesicles (POPC concentration: 0.1 mM) mediated by **1** in the presence of valinomycin. A THF solution of valinomycin (0.005 mol%, 2.5 µL) was added 30 s prior to addition of transporter solution, and the pH gradient dissipation was monitored *via* HPTS fluorescence. At t = 210 s, 50 µL of a Triton X-100 solution (11% in 7:1 water:DMSO (v/v)) was added to lyse the vesicles and fully destroy the pH gradient. The value corresponding to complete pH gradient dissipation was recorded at 270 s, 1 min after lysing the vesicles. EC_{50}(210 s) = 0.0069 ± 0.0007 mol%, n = 0.73 ± 0.05. The same THF control was used in Figures S19-S20.

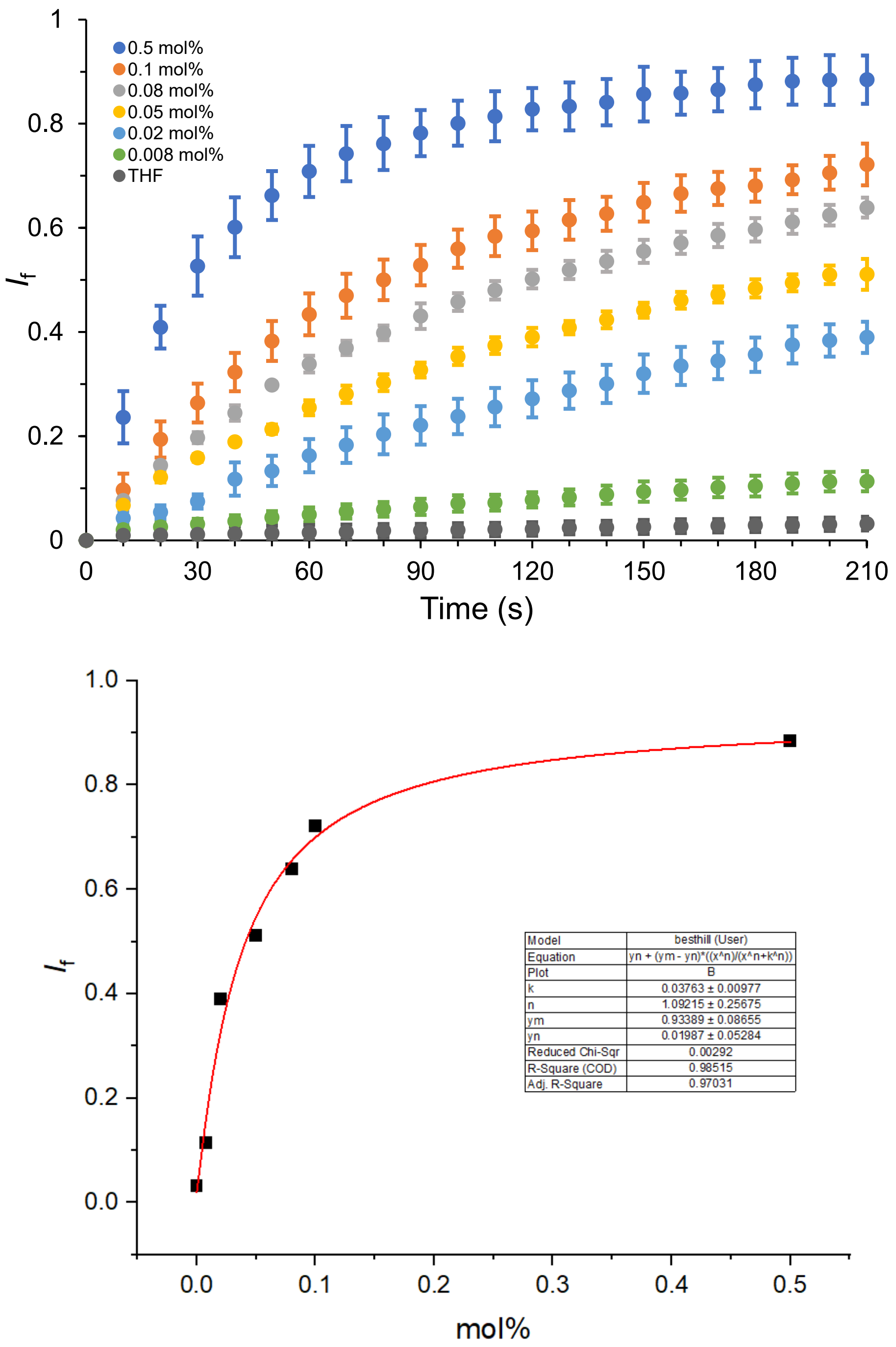

Figure S16. Hill plot analysis of the hydroxide influx into POPC vesicles (POPC concentration: 0.1 mM) mediated by **2** in the presence of valinomycin. A THF solution of valinomycin (0.005 mol%, 2.5 μL) was added 30 s prior to addition of transporter solution (5 μL), and the pH gradient dissipation was monitored *via* HPTS fluorescence. At t = 210 s, 50 μL of a Triton X-100 solution (11% in 7:1 water:DMSO (v/v)) was added to lyse the vesicles and fully destroy the pH gradient. The value corresponding to complete pH gradient dissipation was recorded at 270 s, 1 min after lysing the vesicles. EC_{50}(210 s) = 0.038 ± 0.01 mol%, n = 1.09 ± 0.26. The same THF control was used in Figures S19-S20.

4.4. Carboxyfluorescein (CF) non-specific leakage assay

The following assay was adapted from previous reports.[22] Vesicles loaded with a buffered CF solution (10 mM NaCl, 50 mM CF, 10 mM HEPES, pH 7.4) were added to a fluorescence cuvette contained a buffered saline solution (100 mM NaCl, 10 mM HEPES, pH 7.4) to afford a solution (3 mL) with a final lipid concentration of 0.1 mM. This solution was then transferred into the fluorescence spectrometer, after which it was irradiated at λ_{ex} = 492 nm and the emission intensity was monitored at λ_{em} = 517 nm. The transporter solution (0.6 µL, 2 mol% with respect to lipid concentration) was added at t = 50 s as a DMSO solution. The vesicles were lysed by addition of a detergent solution (50 µL, 11% Triton X-100 in water:DMSO (7:1, v/v)). A final intensity reading was recorded at t = 500 s. The fluorescence intensities I_t was normalized to fractional intensities I_f using the following equation:

$$I_f = \frac{I_t - I_0}{I_\infty - I_0}$$

such that:

I_t = intensity at a given time
$I_0 = I_t$ at t = 50 s
$I_\infty = I_t$ at t = 500 s.

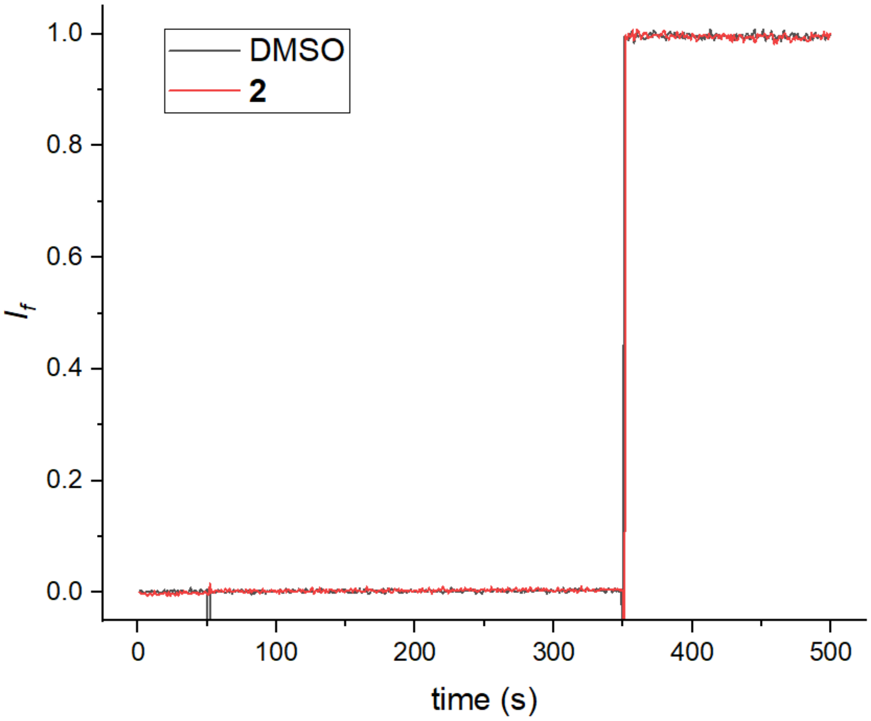

Figure S17. CF non-specific leakage for DMSO (black) and **2** (red). Fluorescence intensity was monitored after the addition of the transporters at 2 mol% concentration at t = 50 s. A detergent solution (7:1:1 H_2O:DMSO:Triton X-100 (v/v/v)) was added at t = 350 s. The sharp discontinuities arise from pausing the fluorescence reading to inject either the transporter or detergent solution into the vesicle solution.

5. References

1. Holmes, R. R.; Day, R. O.; Chandrasekhar, V.; Holmes, J. M., Pentacoordinated Molecules .67. Formation and Structure of Cyclic 5-Coordinated Antimony Derivatives - the 1st Square-Pyramidal Geometry for a Bicyclic Stiborane. *Inorg. Chem.* **1987**, *26*, 157-163.

2. Chan, K. H.; Leong, W. K.; Mak, K. H. G., Thermolysis of the Osmium−Antimony Clusters $Os_3(CO)_{11}(SbMe_2Ar)$: Higher Nuclearity Clusters and Arrested Ortho Metalation. *Organometallics* **2006**, *25*, 250-259.

3. Sobolev, A. N.; Romm, I. P.; Belsky, V. K.; Syutkina, O. P.; Guryanova, E. N., Structure analysis of triaryl derivatives of the group V elements III. Molecular Structure and Spectra of Tris(2,6-Dimethylphenyl)Stibine, $C_{24}H_{27}Sb$. *J. Organomet. Chem.* **1981**, *209*, 49-55.

4. Bruker, 2019, APEX3 (v2019.2011-2010), Bruker AXS Inc., Madison, Wisconsin, USA.

5. Sheldrick, G. M. *SADABS, Version 2007/4*, Bruker Analytical X-ray Systems Inc.: Madison, Wisconsin, USA, 2007.

6. Sheldrick, G. M., SHELXT - integrated space-group and crystal-structure determination. *Acta Crystallogr. A* **2015**, *71*, 3-8.

7. Sheldrick, G. M. *SHELXL-2014: Program for Crystal Structure Refinement*, University of Göttingen, Germany, 2014.

8. Dolomanov, O. V.; Bourhis, L. J.; Gildea, R. J.; Howard, J. A. K.; Puschmann, H., OLEX2: a complete structure solution, refinement and analysis program. *J. Appl. Crystallogr.* **2009**, *42*, 339-341.

9. Frisch, M. J.; Trucks, G. W.; Schlegel, H. B.; Scuseria, G. E.; Robb, M. A.; Cheeseman, J. R.; Scalmani, G.; Barone, V.; Petersson, G. A.; Nakatsuji, H.; Li, X.; Caricato, M.; Marenich, A. V.; Bloino, J.; Janesko, B. G.; Gomperts, R.; Mennucci, B.; Hratchian, H. P.; Ortiz, J. V.; Izmaylov, A. F.; Sonnenberg, J. L.; Williams; Ding, F.; Lipparini, F.; Egidi, F.; Goings, J.; Peng, B.; Petrone, A.; Henderson, T.; Ranasinghe, D.; Zakrzewski, V. G.; Gao, J.; Rega, N.; Zheng, G.; Liang, W.; Hada, M.; Ehara, M.; Toyota, K.; Fukuda, R.; Hasegawa, J.; Ishida, M.; Nakajima, T.; Honda, Y.; Kitao, O.; Nakai, H.; Vreven, T.; Throssell, K.; Montgomery Jr., J. A.; Peralta, J. E.; Ogliaro, F.; Bearpark, M. J.; Heyd, J. J.; Brothers, E. N.; Kudin, K. N.; Staroverov, V. N.; Keith, T. A.; Kobayashi, R.; Normand, J.; Raghavachari, K.; Rendell, A. P.; Burant, J. C.; Iyengar, S. S.; Tomasi, J.; Cossi, M.; Millam, J. M.; Klene, M.; Adamo, C.; Cammi, R.; Ochterski, J. W.; Martin, R. L.; Morokuma, K.; Farkas, O.; Foresman, J. B.; Fox, D. J. *Gaussian 16 Rev. C.01*, Wallingford, CT, 2016.

10. (a) Adams, E. A.; Kolis, J. W.; Pennington, W. T., Structure of triphenylstibine. *Acta Crystallogr., Sect. C: Cryst. Struct. Commun.* **1990**, *46*, 917-919; (b) Sharma, P.; Cabrera, A.; Rosas, N.; Le Lagadec, R.; Hernandez, S.; Valdes, J.; Arias, J. L.; Ambrouse, C. V., Crystal Structures of Tri(*O*-Tolyl)stibine in Two Crystal Forms. *Main Group Met. Chem.* **1998**, *21*, 303-308; (c) Chishiro, A.; Akioka, I.; Sumida, A.; Oka, K.; Tohnai, N.; Yumura, T.; Imoto, H.; Naka,

K., Tetrachlorocatecholates of triarylarsines as a novel class of Lewis acids. *Dalton Trans.* **2022**, *51*, 13716-13724.

11. (a) Becke, A. D., Density-functional thermochemistry. III. The role of exact exchange. *J. Chem. Phys.* **1993**, *98*, 5648-5652; (b) Lee, C. T.; Yang, W. T.; Parr, R. G., Development of the Colle-Salvetti Correlation-Energy Formula into a Functional of the Electron-Density. *Phys. Rev. B* **1988**, *37*, 785-789.

12. (a) Peterson, K. A., Systematically convergent basis sets with relativistic pseudopotentials. I. Correlation consistent basis sets for the post-d group 13--15 elements. *J. Chem. Phys.* **2003**, *119*, 11099-11112; (b) Peterson, K. A.; Figgen, D.; Goll, E.; Stoll, H.; Dolg, M., Systematically convergent basis sets with relativistic pseudopotentials. II. Small-core pseudopotentials and correlation consistent basis sets for the post-d group 16--18 elements. *J. Chem. Phys.* **2003**, *119*, 11113-11123; (c) Peterson, K. A.; Shepler, B. C.; Figgen, D.; Stoll, H., On the Spectroscopic and Thermochemical Properties of ClO, BrO, IO, and Their Anions. *The Journal of Physical Chemistry A* **2006**, *110*, 13877-13883.

13. (a) Petersson, G. A.; Bennett, A.; Tensfeldt, T. G.; Al-Laham, M. A.; Shirley, W. A.; Mantzaris, J., A complete basis set model chemistry. I. The total energies of closed-shell atoms and hydrides of the first-row elements. *J. Chem. Phys.* **1988**, *89*, 2193-2218; (b) Petersson, G. A.; Al-Laham, M. A., A complete basis set model chemistry. II. Open-shell systems and the total energies of the first-row atoms. *J. Chem. Phys.* **1991**, *94*, 6081-6090.

14. (a) Ditchfield, R.; Hehre, W. J.; Pople, J. A., Self-Consistent Molecular-Orbital Methods. IX. An Extended Gaussian-Type Basis for Molecular-Orbital Studies of Organic Molecules. *J. Chem. Phys.* **1971**, *54*, 724-728; (b) Hehre, W. J.; Ditchfield, R.; Pople, J. A., Self-Consistent Molecular Orbital Methods. XII. Further Extensions of Gaussian-Type Basis Sets for Use in Molecular Orbital Studies of Organic Molecules. *J. Chem. Phys.* **1972**, *56*, 2257-2261.

15. Falivene, L.; Cao, Z.; Petta, A.; Serra, L.; Poater, A.; Oliva, R.; Scarano, V.; Cavallo, L., Towards the online computer-aided design of catalytic pockets. *Nat. Chem.* **2019**, *11*, 872-879.

16. Lu, T.; Chen, F., Multiwfn: A multifunctional wavefunction analyzer. *J. Comput. Chem.* **2012**, *33*, 580-592.

17. Dennington, R.; Keith, T. A.; Millam, J. M. *GaussView, Version 6.1.1*, Version 6.1.1; Semichem Inc.: Shawnee Mission, KS, 2019.

18. (a) Ribeiro, R. F.; Marenich, A. V.; Cramer, C. J.; Truhlar, D. G., Use of Solution-Phase Vibrational Frequencies in Continuum Models for the Free Energy of Solvation. *J. Phys. Chem. B* **2011**, *115*, 14556-14562; (b) Kolar, M.; Fanfrlik, J.; Lepsik, M.; Forti, F.; Luque, F. J.; Hobza, P., Assessing the Accuracy and Performance of Implicit Solvent Models for Drug Molecules: Conformational Ensemble Approaches. *J. Phys. Chem. B* **2013**, *117*, 5950-5962.

19. Marenich, A. V.; Cramer, C. J.; Truhlar, D. G., Universal Solvation Model Based on Solute Electron Density and on a Continuum Model of the Solvent Defined by the Bulk Dielectric Constant and Atomic Surface Tensions. *J. Phys. Chem. B* **2009**, *113*, 6378-6396.

20. Vlahovic, F.; Ivanovic, S.; Zlatar, M.; Gruden, M., Density functional theory calculation of lipophilicity for organophosphate type pesticides. *J. Serb. Chem. Soc.* **2017**, *82*, 1369-1378.

21. Wu, X.; Judd, L. W.; Howe, E. N. W.; Withecombe, A. M.; Soto-Cerrato, V.; Li, H.; Busschaert, N.; Valkenier, H.; Pérez-Tomás, R.; Sheppard, D. N.; Jiang, Y.-B.; Davis, A. P.; Gale, P. A., Nonprotonophoric Electrogenic Cl^- Transport Mediated by Valinomycin-like Carriers. *Chem* **2016**, *1*, 127-146.

22. Zhou, B.; Gabbaï, F. P., Redox-controlled chalcogen-bonding at tellurium: impact on Lewis acidity and chloride anion transport properties. *Chem. Sci.* **2020**, *11*, 7495-7500.
